# Co-targeting MRPS7-23 synergistically enhances cisplatin efficacy to suppress nasopharyngeal carcinoma growth and metastasis

**DOI:** 10.7150/ijbs.115523

**Published:** 2026-01-01

**Authors:** Zhangqi Cao, Can Pan, Zeyu Liu, Qi Quan, Mengping Li, Yu Huang, Chuwen Liang, Yuwen Chen, Teng Fan, Ping Chen, Fu Kai, Shuangli Zhu, Sijia Li, Xin Su, Fang Wang, Liwu Fu, Bei Zhang

**Affiliations:** 1 TCM&VIP Department, Sun Yat-sen University Cancer Center, Guangzhou, People's Republic of China.; 2 State Key Laboratory of Oncology in South China, Guangdong Key Laboratory of Nasopharyngeal Carcinoma Diagnosis and Therapy, Guangdong Provincial Clinical Research Center for Cancer, Sun Yat-sen University Cancer Center, Guangzhou, People's Republic of China.; 3 Integrated Traditional Chinese and Western Medicine Research Center, Sun Yat-sen University Cancer Center, Guangzhou, People's Republic of China.; 4 Department of Pharmacy, Sun Yat-sen University Cancer Center, Guangzhou, People's Republic of China.

**Keywords:** MRPS7, MRPS23, nasopharyngeal carcinoma, cisplatin resistance, metastasis

## Abstract

While cisplatin-based chemoradiotherapy regimens (gemcitabine-cisplatin [GP] and docetaxel-cisplatin-5-fluorouracil [TPF]) remain standard treatments for advanced nasopharyngeal carcinoma (NPC), 30-40% of patients exhibit intrinsic chemoresistance, resulting in therapeutic failure. The molecular underpinnings of this resistance are poorly characterized. Through integrative multi-omics profiling, we identified Mitochondrial Ribosomal Protein S7 (MRPS7) and Mitochondrial Ribosomal Protein S23 (MRPS23) as novel drivers of cisplatin resistance in NPC. Mechanistically, integrated single-cell RNA-seq (scRNA-seq) analysis, mass spectrometry, and functional studies revealed that MRPS7 and MRPS23 stabilized β-catenin by inhibiting its ubiquitination, thereby promoting β-catenin-mediated cancer stemness and epithelial-mesenchymal transition (EMT) to establish cisplatin resistance in NPC. Additionally, we identified Ubiquitin Specific Peptidase 10 (USP10) as a critical upstream regulator that protects MRPS7/23 from proteasomal degradation and sustaining their oncogenic activity. Notably, Spautin-1, a potent USP10 inhibitor, demonstrates synergistic therapeutic activity with cisplatin in diminished tumor growth and metastasis in NPC mice. This research established the USP10-MRPS7/MRPS23-β-catenin axis as a promising precision medicine strategy to combat metastatic dissemination and reverse cisplatin chemoresistance in advanced NPC, which offers a promising opportunity to develop cisplatin sensitizers for the clinical translation of NPC therapies.

## Introduction

NPC is a unique form of head and neck squamous cell carcinoma (HNSC), characterized by high malignancy and close link to Epstein-Barr virus (EBV) infection. Its incidence is particularly high in Southeast Asia, creating a critical demand for the discovery of new prognostic biomarkers and treatment approaches to enhance patient outcomes^1^-^3^. The multicenter phase III randomized controlled trial evaluating induction chemotherapy strategies in locoregionally advanced NPC revealed that the GP regimen significantly outperformed the conventional TPF combination in terms of OS, potentially through the eradication of micrometastatic disease^4^, ^5^. Cisplatin-based concurrent/adjuvant radiation and chemotherapy is regarded as the standard treatment for locally advanced NPC and has significantly improved local control of HNSC^6^-^8^. However, cisplatin resistance critically limits therapeutic efficacy in advanced NPC, resulting in unsatisfactory clinical outcomes^8^, ^9^. Increasing evidence shows that the processes of EMT and cancer stemness are closely associated with chemoresistance in nasopharyngeal carcinoma^10^, ^11^. Mechanistically, activation of the Wnt/β-catenin signaling pathway has been shown to promote the maintenance of cancer stem-like properties and enhance EMT^12^, ^13^, thereby contributing to therapy resistance and tumor progression. Therefore, in order to make patients more sensitive to chemotherapy for precise treatment of locoregionally advanced NPC, novel prospective targets, molecular pathogenic mechanisms, and therapeutic approaches are desperately needed.

Mitochondrial ribosomal small subunit proteins (MRPSs), encoded by nuclear genes, play a pivotal role in maintaining mitochondrial translation and cellular energy production, processes that are mechanistically implicated in tumorigenesis, progression, and metastasis^14^, ^15^. Emerging evidence has demonstrated significant associations between MRPSs and various malignancies, including but not limited to adrenal cortical carcinoma, breast cancer, clear cell renal cell carcinoma, bladder cancer, cervical carcinoma, esophageal squamous cell carcinoma, gastric cancer, colorectal cancer, lung cancer, ovarian cancer, pancreatic cancer, glioma, prostate cancer, hepatocellular carcinoma, and schwannoma^16^-^19^. Although MRPSs have been extensively implicated in various malignancies, their precise role in NPC remains poorly characterized in the current. Consequently, it is crucial to elucidate the molecular mechanisms and establish robust evidence supporting the role of MRPSs in advanced NPC progression. MRPS7 has been shown to be highly expressed in breast cancer, where it serves as a critical regulator of metabolic reprogramming in malignant cells^20^. However, the tumor-suppressive effects of MRPS7 downregulation remain poorly understood. MRPS23 has been identified as a novel prognostic biomarker in glioma, adrenal cortical carcinoma, and colorectal cancer^21^-^23^. Elevated MRPS23 expression promotes tumorigenic processes and contributes to paclitaxel resistance in breast cancer^24^. Furthermore, MRPS23 demonstrates diagnostic potential in hepatocellular carcinoma, with implications for prognosis prediction, immune characterization, and drug sensitivity assessment^25^. Given the established oncogenic functions of MRPS7 and MRPS23, it is plausible that their involvement in mitochondrial translation and cellular energy production may be significantly associated with NPC pathogenesis. Nevertheless, the specific contributions of MRPS7 and MRPS23 to NPC tumorigenesis and progression remain to be fully elucidated.

USP10, a prominent member of the ubiquitin-specific protease (USP) family, plays a crucial role in protein homeostasis through its deubiquitinating activity and regulation of protein function^26^-^28^. The tumor-specific biological context significantly influences the diverse functions and regulatory mechanisms of USP10 in tumorigenesis and cancer progression^29^-^33^. In breast cancer, USP10 enhances tumor cell proliferation, stemness, and metastatic potential by deubiquitinating and stabilizing CD44^34^, ^35^. Additionally, targeting the USP10/B7-H4 proteolytic axis has been shown to enhance the therapeutic efficacy of antibody-drug conjugates (ADCs) in immune-cold tumors^36^. The USP10/XAB2/ANXA2 signaling axis orchestrates homologous recombination-mediated DNA damage repair via stabilization of key repair complexes, thereby driving oxaliplatin resistance in preclinical models of colorectal cancer^37^. The interaction between circWSB1 and USP10 has been shown to drive breast cancer progression by facilitating p53 destabilization^38^. USP10 plays an established role in promoting tumor progression in multiple malignancies, such as colorectal cancer, pancreatic ductal adenocarcinoma, osteosarcoma, esophageal squamous cell carcinoma, non-small cell lung cancer, hepatocellular carcinoma, glioblastoma, and prostate cancer^39^-^43^. Emerging evidence in NPC reveals that USP10 inhibition disrupts G3BP1 deubiquitination, mechanistically linked to cisplatin chemoresistance and metastatic dissemination^9^. While some studies have established USP10's tumor-promoting functions across various cancer types, the precise molecular mechanisms underlying USP10's role in NPC pathogenesis remain to be fully elucidated.

Here, our study reveals that MRPS7 and MRPS23 critically stabilize β-catenin by inhibiting its ubiquitination, thereby promoting β-catenin-mediated cancer stemness and EMT in NPC. We further observed that MRPS7 and MRPS23 are significantly overexpressed in NPC, and their suppression potentiates cisplatin's efficacy in inhibiting subcutaneous tumor growth and pulmonary metastasis in NPC xenograft models. Mechanistically, we identified USP10 as a key binding partner of MRPS7 and MRPS23, shielding these proteins from ubiquitin-dependent degradation and sustaining their oncogenic activity. Notably, Spautin-1, a potent USP10 inhibitor, demonstrates synergistic therapeutic activity with cisplatin, offering a promising combinatorial therapeutic approach for NPC patients. Collectively, our mechanistic insights not only identify mitochondrial ribosomal proteins MRPS7 and MRPS23 as critical mediators of NPC pathogenesis but also reveal their dual utility as both prognostic indicators and therapeutic targets. Notably, these findings further propose that pharmacological targeting of the USP10-MRPS7/MRPS23-β-catenin signaling axis represents a promising precision medicine approach to inhibit metastatic dissemination and overcome cisplatin chemoresistance in advanced NPC.

## Materials and Methods

### Data collection and analysis

To systematically investigate the expression profiles of mitochondrial ribosomal proteins (MRPSs) in human cancers, we integrated bulk and single-cell RNA sequencing datasets from multiple public repositories. First, we obtained bulk RNA-sequencing data (transcripts per million normalized) comprising 10,363 primary tumor samples representing 33 cancer types and 730 matched para-cancerous normal tissues from The Cancer Genome Atlas (TCGA) database (https://portal.gdc.cancer.gov). From this collection, we specifically analyzed 519 head and HNSC tumors and 44 adjacent normal mucosa samples as a focused subset. For NPC analysis, we obtained an independent validation dataset from the Gene Expression Omnibus (GEO accession: GSE61218; https://www.ncbi.nlm.nih.gov/geo), comprising 10 primary NPC tumor specimens and 6 normal nasopharyngeal epithelium samples. This carefully matched cohort served as a critical validation set, allowing rigorous comparison of MRPS7 and MRPS23 expression profiles between malignant and benign tissues. To characterize the cellular distribution of MRPS7 and MRPS23 expression within the NPC tumor microenvironment at single-cell resolution, we analyzed two independent single-cell RNA sequencing datasets (NPC-GSE150430 and NPC-GSE162025) obtained from the Tumor Immune Single-Cell Hub (TISCH; http://tisch.comp-genomics.org). This high-resolution approach enabled precise identification of specific cell subpopulations expressing these mitochondrial ribosomal proteins across malignant, stromal, and immune cell compartments within NPC tumors.

### Clinical specimens

A total of 41 paraffin-embedded NPC specimens were retrospectively obtained from patients who had undergone cisplatin-based chemotherapy at Sun Yat-sen University Cancer Center (Guangzhou, China). Treatment response to cisplatin was evaluated according to RECIST 1.1 criteria. In accordance with the Declaration of Helsinki, we obtained written informed consent from all patients prior to specimen collection under an IRB-approved protocol at Sun Yat-sen University Cancer Center. This consent included the use of their clinical data, archival records, and paraffin-embedded tissue specimens for research. All cases were restaged in accordance with the 7th edition of the American Joint Committee on Cancer staging manual^44^. Relevant clinical characteristics of the included patients are summarized in Supplementary Tables 1-3.

### LASSO regression analysis of hub gene selection

To identify key mitochondrial ribosomal protein (MRPS) genes with clinical relevance in HNSC, we employed a least absolute shrinkage and selection operator (LASSO) regression approach. The analysis was conducted using R statistical software (version 4.1.2) with the glmnet package (version 4.1-2) for model implementation and the survival package (version 3.2-10) for prognostic evaluation. From our initial panel of 29 MRPS candidate genes, the LASSO algorithm with 10-fold cross-validation identified six genes (MRPS5, MRPS7, MRPS18A, MRPS22, MRPS23, and MRPS24) that showed significant prognostic value in HNSC.

### Cell lines and culture

The human NPC cell lines used in this study were generously provided by Professor Liwu Fu at Sun Yat-sen University Cancer Center. Specifically, we utilized two nasopharyngeal carcinoma cell lines: C666 (an Epstein-Barr virus-positive cell line) and Cne2 (a poorly differentiated nasopharyngeal carcinoma cell line). All experimental cell lines underwent propagation in RPMI-1640 basal medium (Gibco) enriched with 10% heat-inactivated fetal bovine serum (FBS; Gibco) and 1% penicillin-streptomycin antibiotic cocktail (Sigma-Aldrich). Cellular maintenance was conducted under standard culture conditions (37°C, 5% CO_2_) within a humidity-controlled incubator, with medium changes performed every 48 hours.

### Cell proliferation assay

Cell proliferation was evaluated with the Cell Counting Kit-8 (CCK-8) assay (Dojindo Laboratories, Kumamoto, Japan) following the supplier's recommended standardized protocols. Briefly, C666 and Cne2 cells, including their respective knockdown variants (shMRPS7, shMRPS23, and shMRPS7+23), were plated at 96-well plates and maintained for 24, 48, 72, and 96 hours. At each time point, 10 μL aliquot of CCK-8 reagent was dispensed into individual wells, followed by incubation at 37 °C for 2 hours. Absorbance was measured at 450 nm using a microplate reader (BioTek Instruments, Winooski, VT, USA).

### Transwell assay

Cell migration was assessed using a Transwell chamber assay with 8 μm pore polycarbonate membranes (Corning Inc., Corning, NY, USA). Briefly, Cell suspensions (200 μL) prepared in serum-free RPMI-1640 medium were placed into the upper compartment of migration chambers, while the lower chamber contained 500 μL complete medium supplemented with 10% FBS as a chemoattractant. Following 48 hours of incubation at 37°C under 5% CO_2_, non-migrated cells on the upper membrane surface were carefully removed using cotton swabs. Cells adherent to the lower membrane underwent sequential fixation in 4% paraformaldehyde 15 min, (PFA), 0.1% crystal violet staining 20 min, and three PBS washes to remove unbound dye. Three representative microscopic fields per well were examined at 100× magnification using an inverted phase-contrast microscope (Nikon, Japan), and migrated cells were quantified using ImageJ software (v1.53). All experimental conditions were assessed in triplicate wells and repeated in three independent biological replicates.

### Spheroid formation assay

Single cells of C666 and Cne2 lines, along with their corresponding knockdown variants (shMRPS7, shMRPS23, and shMRPS7+23), were plated in 12-well ultra-low attachment plates and maintained in serum-free DMEM/F12 medium (Gibco) supplemented with 20 ng/mL epidermal growth factor (Invitrogen) and 10 ng/mL basic fibroblast growth factor (Invitrogen). Following a 10-day culture period, spheroid formation was quantified and representative images were captured using microscopy.

### Flow cytometry

For the analysis of the side population (SP), NPC cells and their corresponding knockdown variants (shMRPS7+23) were harvested and stained with Hoechst 33342 dye (5 mg/mL, Sigma) with or without verapamil. The cells were incubated in darkness at 37 °C for 1.5 hours with intermediate mixing. The reaction was halted by placing the samples in an ice bath, followed by two washes with ice-cold PBS. Finally, flow cytometry was performed to analyze the side population.

### Immunofluorescence assay

C666 and Cne2 cells were fixed with 4% paraformaldehyde for 30 min and permeabilized with 0.5% Triton X-100 for 1 h. After blocking with 5% BSA for 1 h, the cells were incubated overnight at 4 °C with the following primary antibodies: USP10 (1:200, Cell Signaling Technology), MRPS7 (1:200, Abcam), MRPS23 (1:200, Proteintech), and β-catenin (1:200, Proteintech). Subsequently, cells were incubated with secondary antibodies (Proteintech) for 1 h at room temperature. Nuclei were counterstained with DAPI (Beyotime Biotechnology), and fluorescence images were acquired using a confocal laser scanning microscope (Nikon, Japan).

### Quantitative Real Time Polymerase Chain Reaction (qRT-PCR)

Total RNA extraction from C666 and Cne2 cell lines was performed employing TRIzol Reagent (Thermo Fisher Scientific, USA) following the manufacturer's protocol. Reverse transcription to synthesize cDNA was conducted using the PrimeScript RT Reagent Kit (Takara, Japan). Quantitative real-time PCR was then performed with SYBR Green Master Mix (Yeasen Biotechnology, China) to determine mRNA expression levels. GAPDH was used as the endogenous control, and relative gene expression was calculated using the 2^-ΔΔCT^ method.

### Co-immunoprecipitation (Co-IP) and western blot

For Co-IP experiments, following two washes with PBS, C666 and Cne2 cellular lysis was performed with RIPA buffer (Beyotime Biotechnology, China) supplemented with dual protease/phosphatase inhibitor cocktails (Beyotime). After 30 min incubation on ice, lysates were collected with chilled scrapers and centrifuged (15,000 × g, 20 min, 4 °C) to obtain clarified supernatants. Protein quantification of the supernatants was performed with the BCA assay kit (Beyotime). A small amount of the cell lysate supernatant was reserved as the input control. The remaining lysate was incubated with 2 μg of the appropriate primary antibody for 24 hours at 4 °C with constant rotation. Subsequently, 50 μL pre-conditioned protein A/G magnetic beads (Beyotime) were combined with the immunocomplexes and rotated at 4°C for 4 h to facilitate antibody-antigen binding. The beads were then pelleted magnetically and washed five times with ice-cold lysis buffer to remove nonspecific binding. Finally, the input samples, negative control (IgG), and immunoprecipitated (IP) fractions were analyzed by western blotting to verify specific protein-protein interactions. For western blot analysis, After SDS-PAGE (10%) and PVDF transfer (Millipore), membranes were blocked (5% milk, 1 h RT) then incubated with primary antibodies (4°C, overnight): MRPS7 (1:1000, Abcam, UK), MRPS23 (1:1000, Proteintech, USA), β-catenin (1:1000, Proteintech, USA), Slug (1:1000, Proteintech, USA), Vimentin (1:1000, Proteintech, USA), E-cadherin (1:1000, Proteintech, USA), USP10 (1:1000, Proteintech, USA), SOX2 (1:1000, Proteintech, USA), Oct4 (1:1000, Abcam, UK), and Nanog (1:1000, Abcam, UK). After washing with PBST, the membranes were incubated with horseradish peroxidase (HRP)-conjugated secondary antibodies (1:5000; Proteintech, USA) for 60 minutes at ambient temperature. Protein signals were detected using an enhanced chemiluminescence (ECL) substrate (Bio-Rad, USA) and visualized with a Tanon 5200 Multi imaging system (Tanon, China).

### LC-MS/MS quantification

Protein samples extracted from Cne2 cells were prepared for quantitative proteomic analysis following standard protocols. Cell lysates prepared in inhibitor-supplemented RIPA buffer were quantified using the BCA assay, and equal protein loads were electrophoresed (10% SDS-PAGE) for Coomassie staining.

Each lane was systematically excised into 1 mm^3^ gel slices, which were then subjected to in-gel tryptic digestion. Briefly, gel fragments were sequentially processed through destaining, reduction using 10 mM DTT, alkylation with 55 mM iodoacetamide, and overnight proteolysis with trypsin (Promega, sequencing-grade). Purified peptides were desalted with C18 StageTips (Thermo Scientific) and eluted in 0.1% formic acid prior to LC-MS/MS analysis. Chromatographic separation was achieved using an Easy-nLC 1200 UHPLC system interfaced with a Q Exactive Plus Orbitrap mass spectrometer (both Thermo Scientific). Nanoflow liquid chromatography was performed using an Easy-nLC 1200 system (Thermo Fisher Scientific) with mobile phase A (0.1% formic acid in water) and phase B (0.1% formic acid in 80% acetonitrile). Separated analytes were directly introduced into a Q Exactive Plus hybrid quadrupole-Orbitrap mass spectrometer (Thermo Fisher Scientific, Waltham, MA) for data acquisition, followed by raw file conversion and processing using Proteome Discoverer software.

### Molecular docking

Molecular docking, a well-established computational method for predicting ligand-protein interactions, was employed to characterize the binding between USP10 and the mitochondrial ribosomal proteins MRPS7 and MRPS23. The crystal structures of MRPS7, MRPS23, and USP10 were obtained from the Protein Data Bank (https://www.rcsb.org/). Docking simulations were performed using AlphaFold3, and the resulting binding poses were visualized and analyzed using PyMOL 1.8.

### Surface plasmon resonance

Surface plasmon resonance (SPR) binding assays were conducted in triplicate at 25 °C on a BIAcore 1K system using CM5 sensor chips (Cytiva). Following chip activation with a mixture of 200 μM 1-ethyl-3-(3-dimethylaminopropyl) carbodiimide (EDC) and 50 μM N-hydroxysuccinimide (NHS) at a flow rate of 10 μL/min for 10 min, USP10 (50 μg/mL in 10 mM sodium acetate, pH 4.0) was immobilized onto the sensor surface via two consecutive injections (10 μL/min, 5 min each). The surface was then blocked with 1 M ethanolamine (10 μL/min, 10 min). A neighbouring cell that served as a reference was similarly activated and blocked, except that PBS adjusted to pH 4.0 was used for immobilization. Both of the cells were then equilibrated with PBS. Stock solutions of MRPS7 and MRPS23 (2 mM stock solutions; Hubei Ipodix Biotechnology Co., Ltd, China) were serially diluted in PBS to generate concentration gradients. For MRPS7, the concentrations were 1, 0.5, 0.25, 0.125, 0.0625 and 0.03125 μM; For MRPS23, the concentrations were 10, 5, 2.5, 1.25 0.625 and 0.3125 μM. Each analyte was injected over the sensor surface at a flow rate of 30 μL/min for 150 s during the binding phase. After each analyte injection, the surface was regenerated with 10 mM glycine-HCl at a flow rate of 10 μL/min for 5 min. Response data were collected via Biacore Insight Software (v. 2.0, Cytiva), reference-subtracted, and globally fitted to a 1:1 Langmuir model using BIAcore 1K Evaluation Software to derive the binding kinetics. Final figures were generated in Origin 7 (v. 7.0552, OriginLab).

### Animal studies

Female immunodeficient BALB/c nude mice (5 weeks old) were purchased from GemPharmatech Co., Ltd (China) and housed under specific pathogen-free conditions at the Sun Yat-sen University Cancer Center animal center. All animal experimental protocols were approved by the Committee on the Ethics of Animal Experiments at Sun Yat-sen University Cancer Center.

For subcutaneous tumor studies, mice were inoculated in the left flank with Cne2 or C666 cells stably expressing the indicated shRNAs. To investigate the impact of MRPS7 and MRPS23 on tumor growth, mice were injected subcutaneously with 1×10^6^ Cne2 cells transfected with shRNA-control, shMRPS7, shMRPS23, or shMRPS7+23. To verify the effects of MRPS7 and MRPS 23 on the stemness, serial dilutions of C666 cells (1×10^6^, 5×10^5^, 1×10^5^, 5×10^4^) transfected with shRNA-control or shMRPS7+23 were implanted subcutaneously. To determine the role of USP10, mice were injected with 1×10^6^ Cne2 cells expressing shRNA-control or shUSP10. For therapeutic assessment of cisplatin resistance, mice were inoculated with 1×10^6^ Cne2 cells and randomized into eight experimental groups: (1) sh-control, (2) shMRPS7, (3) shMRPS23, (4) shMRPS7+23, (5) cisplatin alone, (6) cisplatin + shMRPS7, (7) cisplatin + shMRPS23, and (8) cisplatin + shMRPS7+23. Cisplatin (4 mg/kg) or vehicle control (0.9% saline) was administered intraperitoneally every 72 hours. To further evaluate combined therapeutic effects of Spautin-1 and cisplatin, mice bearing Cne2 xenografts were randomly assigned to the following treatment groups: (1) Control, (2) Spautin-1, (3) Cisplatin, and (4) Spautin-1 + Cisplatin. Mice received daily intraperitoneal injections of either vehicle control, Spautin-1 (20 mg/kg per day), cisplatin (4 mg/kg q3d), Spautin-1 (20 mg/kg per day) + cisplatin (4 mg/kg q3d). Tumor growth was monitored via digital caliper measurements, and volume was calculated using the formula: V = (length × width^2^)/2. Mice were euthanized at the experimental endpoint for tumor excision and weight measurement.

For metastasis studies, mice were administered via tail vein injection with Cne2 cells stably expressing the indicated shRNAs. To investigate the impact of MRPS7 and MRPS23 on tumor metastasis, 5×10^5^ Cne2 cells transduced with shRNA-control, shMRPS7, shMRPS23, or shMRPS7+23 in 150 μL PBS was administered via tail vein injection. For therapeutic assessment of cisplatin resistance, mice injected with 5×10^5^ Cne2 cells were randomly allocated into four treatment groups: (1) sh-control + saline, (2) sh-MRPS7+23 + saline, (3) sh-control + cisplatin, and (4) sh-MRPS7+23 + cisplatin. Beginning 24 hours post-injection, mice received intraperitoneal injections of either cisplatin (4 mg/kg) or equivalent volumes of physiological saline every 3 days. To further evaluate combined therapeutic effects of Spautin-1 and cisplatin, mice were randomly allocated into four treatment groups: (1) Control, (2) Spautin-1, (3) Cisplatin, and (4) Spautin-1 + cisplatin. Beginning 24 hours post-injection, mice were subjected to intraperitoneal administration of either vehicle, Spautin-1 (20 mg/kg per day), Cisplatin (4 mg/kg q3d) or Spautin-1 (20 mg/kg per day) + cisplatin (4 mg/kg q3d). Following experimental completion, euthanized mice underwent systematic lung resection for metastatic nodule quantification. Paraffin-embedded tissue specimens were sectioned at 5 μm thickness and subjected to hematoxylin & eosin (H&E) histological staining.

### Immunohistochemical (IHC) analysis

Xenograft tumor tissues were fixed, paraffin-embedded, and sectioned at a thickness of 4 μm with a rotary microtome. The sections were deparaffinized in xylene and rehydrated through a graded ethanol series. Antigen retrieval was carried out in citrate buffer (pH 6.0) at 95 °C for 20 minutes using a decloaking chamber. After blocking with 5% BSA, the sections were incubated with primary antibodies at 4 °C overnight. Detection was performed using HRP-conjugated secondary antibodies (1:500 dilution) with DAB chromogenic substrate. Whole-slide images were acquired using a digital slide scanner (Olympus, Japan).

### Statistical analysis

Statistical analyses were performed using R software (version 4.2.1; The R Foundation for Statistical Computing, Vienna, Austria), IBM SPSS Statistics (version 22.0; IBM Corp., Armonk, NY, USA), and GraphPad Prism (version 9.0.0; GraphPad Software, Boston, MA, USA). Differences between groups were assessed using Student's t-test or one-way ANOVA, with a *p*-value < 0.05 considered statistically significant.

## Results

### The expression and prognosis of the hub genes of MRPSs

**Figure 1:**
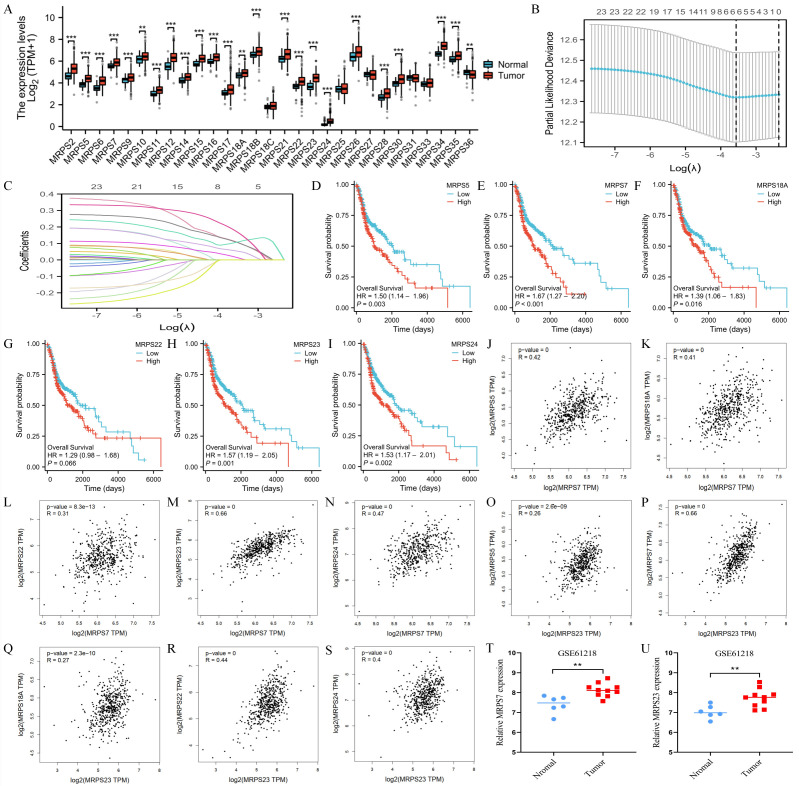
The expression and prognosis of MRPSs hub genes. (A) Differential expression analysis of 29 MRPS family members between HNSC tumors (n=504) and normal tissues (n=44) from TCGA cohort. (B-C) LASSO regression coefficient profiles and feature selection identifying 6 hub genes from 29 MRPS genes. (D-I) Kaplan-Meier survival curves were generated to compare low and high expressed MRPS5, MRPS7, MRPS18A, MRPS22, MRPS23, and MRPS24 in HNSC based on TCGA database. (J-S) Spearman correlation matrices illustrating co-expression patterns among MRPS5/7/18A/22/23/24 in HNSC based on TCGA database. (T-U) Validation of MRPS7 and MRPS23 overexpression in nasopharyngeal carcinoma (NPC) tumors (n = 10) compared with matched normal nasopharyngeal tissues (n = 6) from the GSE61218 dataset. **p < 0.01 and ***p < 0.001.

To systematically investigate the functional significance of mitochondrial ribosomal small subunit proteins in HNSC, we first conducted a comprehensive expression analysis of all 29 MRPS family members using TCGA data (Fig. 1A). Leveraging Lasso regression analysis, we identified MRPS5, MRPS7, MRPS18A, MRPS22, MRPS23, and MRPS24 as hub genes within the MRPS family, suggesting their critical involvement in HNSC pathogenesis (Fig. 1B-C). Subsequent Kaplan-Meier survival analysis indicated that elevated expression of these six MRPS members was significantly associated with poor OS in HNSC patients (Fig. 1D-I), implicating their roles in driving tumor progression and aggressive clinical behavior. Intriguingly, correlation analysis uncovered a robust positive relationship between MRPS7 and MRPS23 (R = 0.66), which was substantially stronger than correlations observed among other MRPS members (Fig. 1J-S). In the meanwhile, we observed that both MRPS7 and MRPS23 were significantly overexpressed in NPC (Fig. 1T-U), further supporting their potential roles in promoting tumor progression across relevant malignancies. Taken together, these findings suggest that MRPS7 and MRPS23 may serve as effective prognostic biomarkers for HNSC and warrant further investigation in the context of NPC.

### MRPS7 and MRPS23 promote nasopharyngeal carcinoma progression *in vitro* and *in vivo*

**Figure 2:**
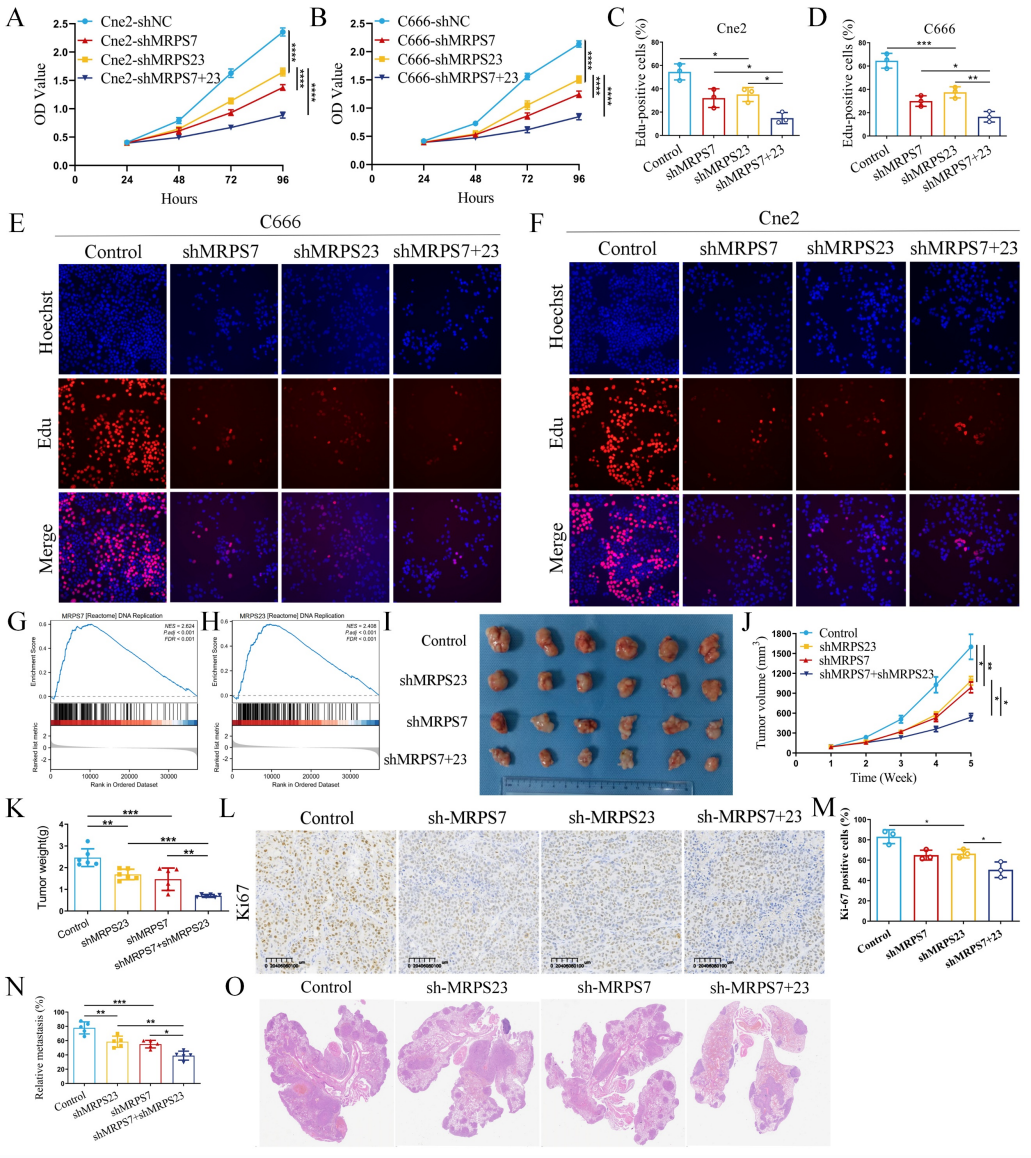
MRPS7 and MRPS23 drive nasopharyngeal carcinoma progression *in vitro* and *in vivo*. (A-B) CCK-8 assays indicated the effect of combined or individual knockdown of MRPS7 and MRPS23 on NPC cell proliferation, compared with control cells. (C-F) EdU assays tested the cooperative role in DNA replication, with dual knockdown MRPS7 and MRPS23 or individual depletions in NPC. (G-H) GSEA analysis revealed the correlation between MRPS7 or MRPS23 and DNA replication pathway activation. (I) Representative images of excised tumors from nude mice bearing NPC xenografts following treatment with: dual MRPS7/MRPS23 knockdown, single knockdown of MRPS7 or MRPS23, scramble control. (J) Tumor volumetric measurements were evaluated at predetermined intervals. (K) Tumor xenograft weights were quantitatively analyzed at endpoint in euthanized mice. (L-M) Ki67 staining and its expression quantification in tumor tissue sections from the xenografts. (N) Quantification of lung metastasis incidence and metastatic nodule counts in each group. (O) Representative H&E staining images of lung tissues from mice injected with NPC cells expressing scramble control, single knockdown of MRPS7 or MRPS23, and dual MRPS7/MRPS23 knockdown. *p < 0.05, **p < 0.01, ***p < 0.001, and ****p < 0.0001.

The significant upregulation of MRPS7 and MRPS23 in NPC led us to investigate the functional role of MRPS7 and MRPS23 in driving the progression of this disease. To delineate the role of MRPS7 and MRPS23 in promoting tumor progression and their potential functional interplay in NPC, we generated MRPS7 and MRPS23 single- and double-knockdown NPC cell lines in the Cne2 and C666 models (Fig. S1A-B, S1G). Functional assessment via CCK-8 assays showed that MRPS7 and MRPS23 ablation suppressed NPC cell growth (Fig. S1C-F). Furthermore, CCK-8 assays confirmed a synergistic inhibitory effect on proliferation in double-knockdown cells compared to single knockdowns (Fig. 2A-B). Functional validation via EdU assays confirmed a cooperative role in DNA replication, with dual knockdown showing greater suppression than individual depletions (Fig. 2C-F). GSEA analysis demonstrated that MRPS7 and MRPS23 expression profiles significantly correlate with DNA replication pathway activation (Fig. 2G-H). To evaluate the *in vivo* relevance of these findings, we employed a subcutaneous xenograft model. Results showed that knockdown of MRPS7 or MRPS23 alone significantly reduced tumor size and weight, while combined knockdown exerted a more pronounced inhibitory effect on tumor growth (Fig. 2I-K). Immunohistochemical analysis revealed lower Ki67 staining in tumors with MRPS7 and MRPS23 knockdown, consistent with reduced proliferative activity (Fig. 2L-M). Furthermore, we established a mouse lung metastasis model via tail vein injection. Knockdown of either MRPS7 or MRPS23 alone significantly reduced lung metastasis, while combined knockdown exerted an even more pronounced inhibitory effect on pulmonary metastatic burden (Fig. 2N-O). Collectively, these findings demonstrate that MRPS7 and MRPS23 function as tumor promoters in NPC, with combined knockdown exhibiting synergistic inhibition of tumor progression and metastasis in NPC.

### The scRNA-seq analysis of MRPS7 and MRPS23 in NPC

**Figure 3:**
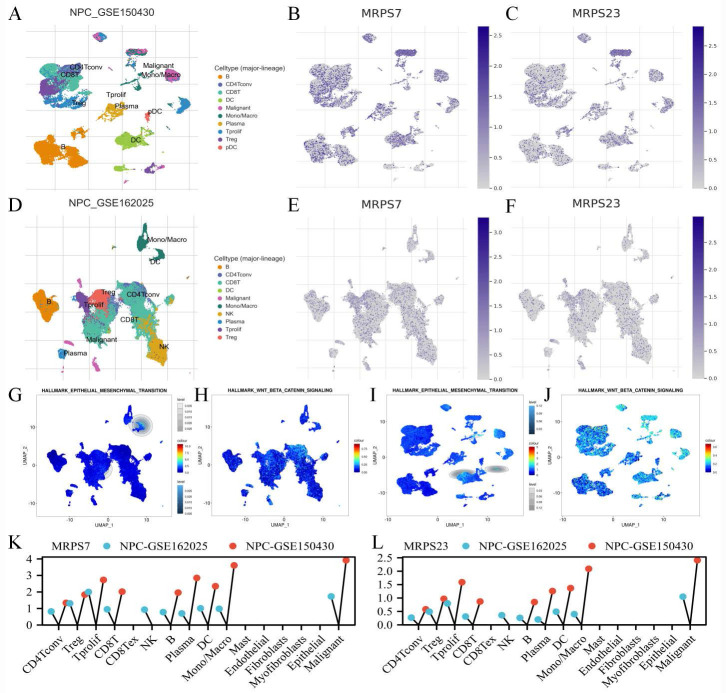
The scRNA-Seq analysis of MRPS7 and MRPS23 in NPC. (A) The t-SNE visualization of single-cell RNA sequencing data from NPC_GSE150430. (B) MRPS7 expression profile across single-cell clusters in NPC_GSE150430. (C) MRPS23 expression profile across single-cell clusters in NPC_GSE150430. (D) The t-SNE visualization of single-cell RNA sequencing data from NPC_GSE162025. (E) MRPS7 expression profile across single-cell clusters in NPC_GSE162025. (F) MRPS23 expression profile across single-cell clusters in NPC_GSE162025. (G) GSEA identified significant enrichment of EMT pathway genes in MRPS7/23-high expressing malignant cells from NPC_GSE162025. (H) GSEA identified significant enrichment of beta-catenin pathway genes in MRPS7/23-high expressing malignant cells from NPC_GSE162025. (I) GSEA revealed positive correlation between MRPS7/23 expression and the Hallmark EMT pathway in scRNA-Seq data of NPC_GSE150430. (J) GSEA revealed positive correlation between MRPS7/23 expression and the Hallmark Wnt-β-catenin pathway in scRNA-Seq data of NPC_GSE150430. (K) Cells enrichment of MRPS7 expression in scRNA-Seq data of NPC_GSE162025 and NPC_GSE150430. (L) Cells enrichment of MRPS23 expression in scRNA-Seq data of NPC_GSE162025 and NPC_GSE150430.

To delineate the specific cell populations expressing MRPS7 and MRPS23 within the tumor microenvironment of NPC, we performed single-cell RNA sequencing (scRNA-seq) analysis using datasets NPC-GSE150430 and NPC-GSE162025 from the TISCH database (Fig. 3A-F). t-Distributed stochastic neighbor embedding (t-SNE) analysis revealed 10 distinct cell clusters across both datasets, In the NPC-GSE150430 dataset, MRPS7 and MRPS23 were predominantly expressed in malignant cells (Fig. 3K-L). This expression pattern was consistently validated in the NPC_GSE162025 dataset (Fig. 3K-L). Integrative analysis of Single-cell transcriptomic profiling uncovered substantial and consistent enrichment of EMT and Wnt/β-catenin signaling pathways specifically in malignant cells exhibiting high expression of MRPS7 and MRPS23 within the NPC_GSE162025 dataset (Fig. 3G-H), providing strong evidence for their functional involvement in regulating these critical oncogenic pathways. Importantly, validation through cross-dataset single-cell transcriptomic profiling of the NPC_GSE150430 cohort confirmed the coordinated activation of both EMT and Wnt/β-catenin signaling in MRPS7/23-overexpressing tumor cells (Fig. 3I-J), defining these mitochondrial ribosomal proteins as dual master regulators of metastatic-inductive signaling programs during nasopharyngeal carcinoma. Collectively, these findings demonstrate that MRPS7 and MRPS23 functionally regulate EMT and Wnt/β-catenin signaling pathways, thereby driving NPC progression.

### MRPS7 and MRPS23 synergistically drive epithelial-mesenchymal transition and cancer stemness in NPC

**Figure 4:**
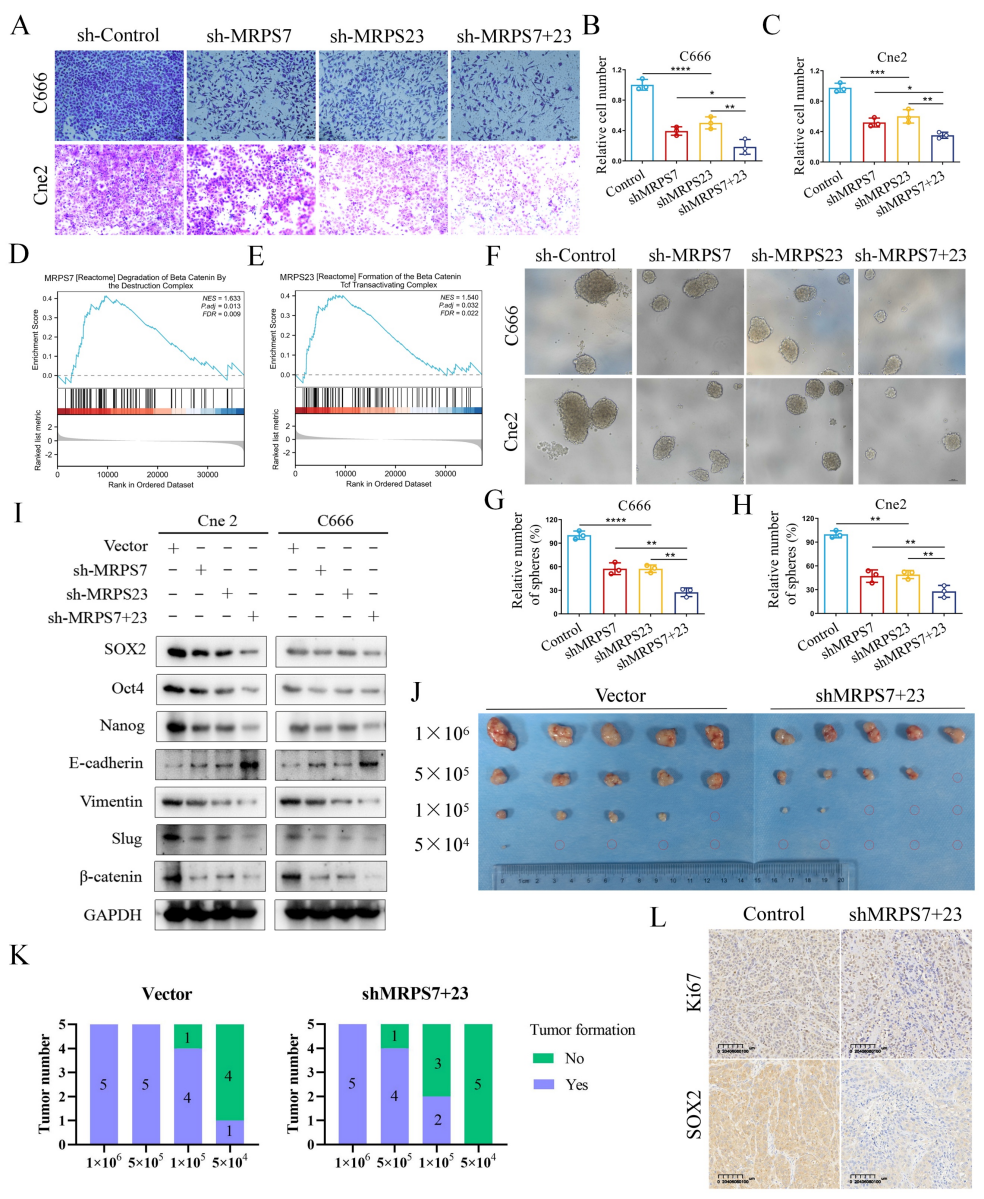
MRPS7 and MRPS23 synergistically promote epithelial-mesenchymal transition and cancer stemness in NPC. (A-C) Transwell migration assays in NPC cells with dual or individual knockdown of MRPS7 and MRPS23. (D) GSEA plot demonstrating significant enrichment (NES=1.633, FDR q=0.009) of the "Degradation of beta-catenin by the destruction complex" gene set with high expression of MRPS7. (E) GSEA plot demonstrating significant enrichment (NES=1.540, FDR q=0.022) of the "Formation of beta-catenin tcf transactiviting complex" gene set in with high expression of MRPS23. (F-H) Representative images of tumorsphere formation assays tested the stemness of NPC cells with dual or individual knockdown of MRPS7 and MRPS23. (I) Western blot analysis showed the protein expression level of SOX2, Oct4, Nanog, E-cadherin, Vimentin, slug and β-catenin in Cne2 and C666 cells with dual knockdown MRPS7 and MRPS23 or individual depletions. (J) The photo of excised C666 tumors. (K) Statistical analysis of tumor formation at different inoculated cell numbers. (L) Ki67 and SOX2 staining and expression of tumor tissue. *p < 0.05, **p < 0.01, ***p < 0.001, and ****p < 0.0001.

Initial scRNA-Seq profiling revealed MRPS7 and MRPS23 as potential regulators of EMT in NPC (Fig. 3G, 3I). Supporting this, the GSEA further demonstrated significant enrichment of the "Degradation of beta-catenin by the destruction complex" gene set in with high expression of MRPS7, concomitant with pronounced activation of the 'β-catenin/TCF transactivation complex formation' pathway in MRPS23-overexpressing samples (Fig. 4D-E). Based on these findings, we performed systematic functional studies to delineate the roles of MRPS7 and MRPS23 in metastatic progression.

The transwell migration assays demonstrated that genetic knockdown of either MRPS7 or MRPS23 significantly attenuated NPC cell migration (Fig. 4A-C). Intriguingly, combinatorial knockdown of both MRPS7 and MRPS23 demonstrated synergistic suppression of migratory capacity (Fig. 4A-C), implying potential functional cooperation between these mitochondrial ribosomal proteins in regulating cellular motility. At the molecular level, western blot analysis of EMT markers revealed that MRPS7 and MRPS23 knockdown significantly upregulated the epithelial marker E-cadherin while downregulating mesenchymal markers Vimentin and Slug (Fig. 4I). Notably, the combinatorial knockdown demonstrated synergistic effects on these molecular alterations (Fig. 4I), corroborating the functional synergy observed in our migration assays. To elucidate the upstream regulatory mechanisms, we performed GSEA pathway analysis, which identified a significant association between MRPS7/MRPS23 expression and β-catenin signaling activation (Fig. 4D-E). This discovery led us to investigate their potential involvement in cancer stemness regulation. Subsequent functional assays demonstrated that MRPS7 and MRPS23 knockdown significantly impaired sphere-forming capacity, as evidenced by reduced sphere size and number (Fig. 4F-H). Western blot analysis further confirmed the downregulation of key stemness markers, including SOX2, Oct4, and Nanog following MRPS7 and MRPS23 knockdown (Fig. 4I). Importantly, the simultaneous knockdown of both genes resulted in a more pronounced reduction in these stemness markers (Fig. 4I), with enhanced coordinated regulation of molecular markers in the dual knockdown condition, consistent with our observations in migration and stemness assays.

Furthermore, to further validate the impact of MRPS7/MRPS23 knockdown on the stemness of nasopharyngeal carcinoma cells, we observed that knockdown of MRPS7 or MRPS23 markedly reduced the proportion of side population (SP) cells via flow cytometry (Fig. S3A-B). Consistently, in a limiting dilution tumorigenicity assay, varying numbers of cells (ranging from 1 × 10^6^ to 5 × 10^4^) were subcutaneously inoculated into nude mice. Both tumor size and tumor-initiating capacity were significantly diminished in the MRPS7/MRPS23 knockdown groups (Fig. 4J-K). Immunohistochemical analysis further revealed a marked reduction in the expression of Ki-67 and the stemness marker SOX2 in tumors derived from the knockdown cells (Fig. 4L). Collectively, our findings reveal that MRPS7 and MRPS23 serve as critical cooperative regulators in the progression of NPC progression, orchestrating a network of oncogenic processes that encompass cellular migration, EMT, and the maintenance of cancer stemness.

### MRPS7 and MRPS23 promote nasopharyngeal carcinoma progression via β-catenin-activated EMT and cancer stemness

**Figure 5:**
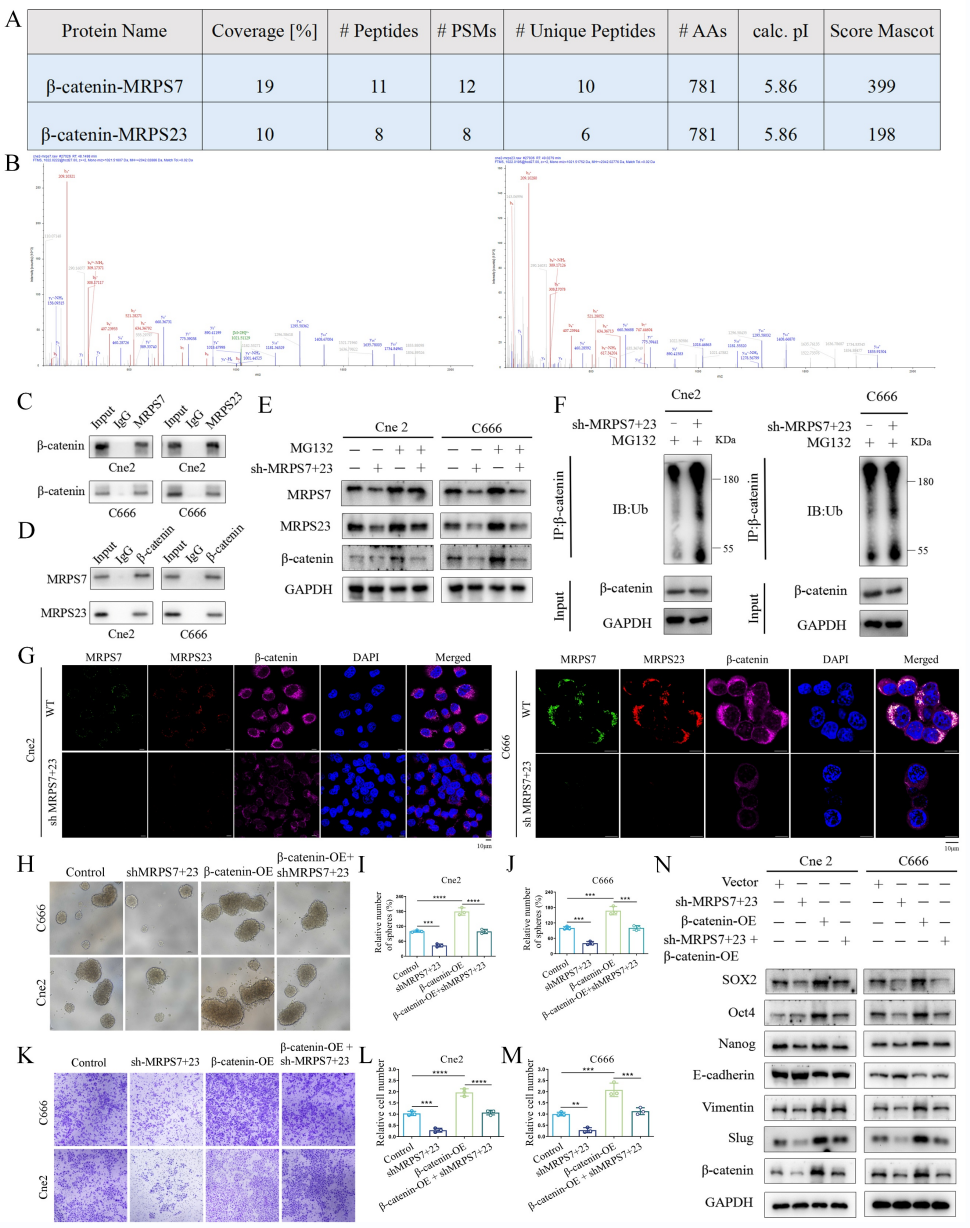
MRPS7 and MRPS23 promote nasopharyngeal carcinoma progression through β-catenin-activated EMT and cancer stemness. (A) The protein of β-catenin co-immunoprecipitated with MRPS7 and MRPS23 was verified by mass spectrometry. (B) Mass spectrometric analysis identified representative peptides of MRPS7 and MRPS23 in β-catenin immunoprecipitates. (C) Co-immunoprecipitation (Co-IP) assays demonstrating β-catenin interaction with MRPS7 and MRPS23 in NPC cells. (D) Reciprocal Co-IP confirming MRPS7/MRPS23 interaction with β-catenin in NPC cells. (E) Western blot analysis of MRPS7, MRPS23, and β-catenin protein levels in NPC cells treated with: scramble control, dual knockdown of MRPS7 and MRPS23, proteasome inhibitor MG132, dual knockdown of MRPS7 and MRPS23 combined with proteasome inhibitor MG132. (F) Western blot analysis of the total and ubiquitinated β-catenin levels in NPC cells with or without dual knockdown of MRPS7 and MRPS23. (G) Immunofluorescence of NPCs with or without dual knockdown of MRPS7 and MRPS23 for MRPS7 (green), MRPS23 (red), β-catenin (purple) and DAPI (blue). The scale bar is 10 μm. (H-J) Representative images of tumorsphere formation assays tested the stemness of NPC cells treated with scramble control, dual knockdown of MRPS7 and MRPS23, β-catenin overexpression, dual knockdown of MRPS7 and MRPS23 combined with β-catenin overexpression. (K-M) Representative images of migrated NPC cells treated with scramble control, dual knockdown of MRPS7 and MRPS23, β-catenin overexpression, dual knockdown of MRPS7 and MRPS23 combined with β-catenin overexpression. (N) Western blot showed the protein expression level of SOX2, Oct4, Nanog, E-cadherin, Vimentin, slug and β-catenin treated in NPC cells with scramble control, dual knockdown of MRPS7 and MRPS23, β-catenin overexpression, dual knockdown of MRPS7 and MRPS23 combined with β-catenin overexpression. **p < 0.01, ***p < 0.001, and ****p < 0.0001.

To elucidate the molecular mechanisms by which MRPS7 and MRPS23 drive NPC progression, we performed co-IP combined with mass spectrometry to identify potential regulatory partners of MRPS7 and MRPS23. IP-MS analysis identified β-catenin as a high-confidence interactor with both MRPS7 and MRPS23, supported by its robust Mascot score, extensive peptide coverage, and a high number of unique peptides (Fig. 5A-B). This interaction was further confirmed by co-IP assays, confirming the binding of β-catenin to both MRPS7 and MRPS23 in NPC cells (Fig. 5C-D). To explore the functional consequences of this interaction, we examined the impact of MRPS7 and MRPS23 on β-catenin expression. Notably, knockdown of MRPS7 and MRPS23 led to a significant reduction in β-catenin protein levels (Fig. 5E), whereas β-catenin mRNA expression remained unaltered under knockdown conditions (Fig. S2A-B). These data demonstrate that MRPS7 and MRPS23 modulate β-catenin levels, potentially via post-translational mechanisms. To elucidate the mechanistic basis of β-catenin regulation by MRPS7 and MRPS23, we evaluated the potential involvement of the ubiquitin-proteasome system. Notably, pharmacological inhibition of proteasomal activity with MG132 markedly attenuated the reduction in β-catenin levels induced by dual knockdown of MRPS7 and MRPS23 in NPC cells (Fig. 5E). Accordingly, dual knockdown of MRPS7 and MRPS23 significantly enhanced the ubiquitination of β-catenin (Fig. 5F). Immunofluorescence analysis further demonstrated that depletion of MRPS7 and MRPS23 resulted in substantial downregulation of β-catenin expression in NPC cells (Fig. 5G). Collectively, these results indicate that MRPS7 and MRPS23 modulate β-catenin stability by regulating its ubiquitin-mediated proteasomal degradation.

Given this regulatory axis, we systematically investigated whether β-catenin serves as the central effector linking MRPS7/MRPS23 depletion to EMT induction and stemness maintenance. Functional rescue experiments demonstrated that β-catenin overexpression significantly restored the migratory capacity of MRPS7/MRPS23-deficient Cne2 and C666 cells in transwell assays (Fig. 5K-M). Additionally, β-catenin reconstitution effectively rescued the impaired tumor sphere formation, as quantified by both sphere size and number (Fig. 5H-J). Molecular profiling revealed a coordinated reversal of EMT and stemness phenotypes: β-catenin overexpression (1) suppressed epithelial characteristics through E-cadherin induction, (2) enhanced mesenchymal transition markers (Vimentin and Slug), and (3) attenuated the expression of core stemness regulators (SOX2, Oct4, and Nanog) (Fig. 5N). Collectively, these findings position MRPS7 and MRPS23 as critical upstream modulators that orchestrate β-catenin-dependent control of two fundamental oncogenic programs in NPC: EMT progression and cancer stem cell maintenance.

### MRPS7 and MRPS23 depletion potentiates cisplatin chemoresponse and suppresses tumorigenesis in NPC

**Figure 6:**
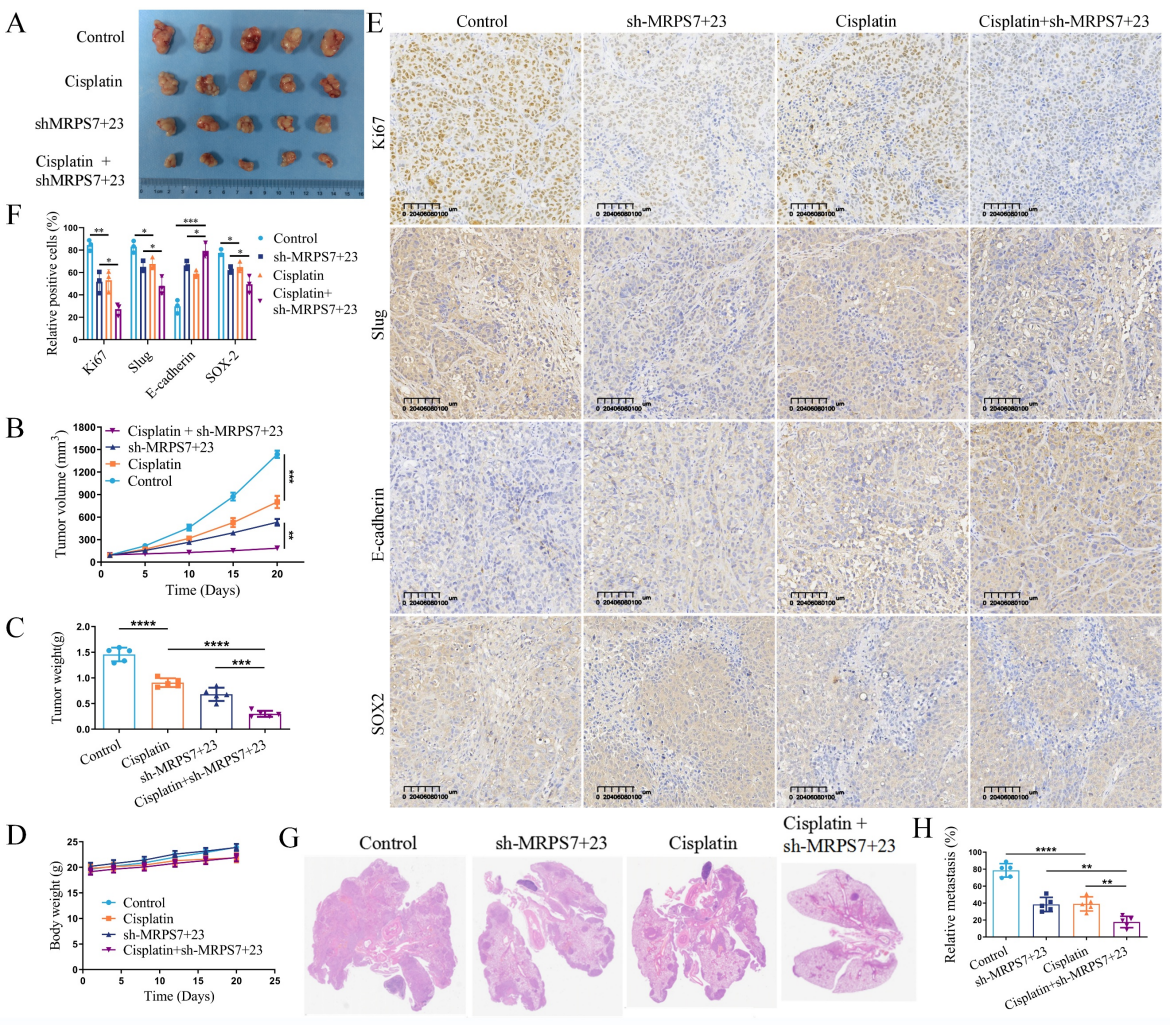
Targeting MRPS7 and MRPS23 potentiates cisplatin chemoresponse and suppresses tumorigenesis in NPC. (A) The photo of excised Cne2 tumors. (B) Tumor volumetric measurements were evaluated at predetermined intervals. (C) The measurement of tumor weight in excised Cne2 tumors (D) Body weight measurements in mice were assessed at predetermined time points. (E) Ki67, slug, E-cadherin and SOX2 staining and expression of tumor tissue. (F) The relative expression of Ki67, slug, E-cadherin and SOX2 in tumor tissue. (G-H) H&E-stained lung tissue of lung metastasis model, with subsequent quantification of microscopically detectable metastatic foci. *p < 0.05, **p < 0.01, ***p < 0.001, and ****p < 0.0001.

Given the critical role of EMT and cancer stemness in mediating chemoresistance and metastatic dissemination, we sought to further elucidate the functional contributions of MRPS7 and MRPS23 to these clinically relevant phenotypes. To this end, we employed subcutaneous xenograft and mouse lung metastasis models. Using subcutaneous tumor xenografts, knockdown of MRPS7 and MRPS23 dramatically potentiated cisplatin efficacy, leading to substantial decreases in both tumor volume and mass in cisplatin-administered murine models. Genetic silencing of MRPS7/MRPS23 decreased tumor burden by 54.13% versus controls. Strikingly, combination with cisplatin synergistically enhanced this antitumor effect, achieving an 82.39% reduction in tumor weight relative to controls (Fig. 6A-C), while maintaining stable body weights in treated mice (Fig. 6D). Furthermore, the results demonstrated that dual knockdown of MRPS7 and MRPS23, combined with cisplatin treatment, resulted in superior antitumor efficacy compared to individual knockdown of either gene alongside cisplatin (Fig. S4). Immunohistochemical analysis further revealed a significant decrease in Ki-67, Slug and SOX2 expression, accompanied by an increase in E-cadherin levels (Fig. 6E-F), suggesting a reversal of EMT coupled with attenuation of cancer cell stemness and reduced proliferative activity. These results corroborate the well-characterized function of EMT and cancer cell stemness in promoting chemoresistance and tumor aggressiveness. Furthermore, in the lung metastasis model, knockdown of MRPS7 and MRPS23 alone led to a deep decrease in the number of metastatic lung lesions. Notably, the combination of MRPS7/MRPS23 knockdown with cisplatin treatment resulted in an even more pronounced decrease in metastatic lung nodules (Fig. 6G-H). These findings further underscore the critical role of MRPS7 and MRPS23 in facilitating metastatic dissemination. Collectively, these findings demonstrate that MRPS7 and MRPS23 play critical roles in promoting cisplatin resistance and NPC progression *in vivo*.

### Depletion of USP10 downregulates MRPS7 and MRPS23 expression and suppresses xenograft tumor growth

**Figure 7:**
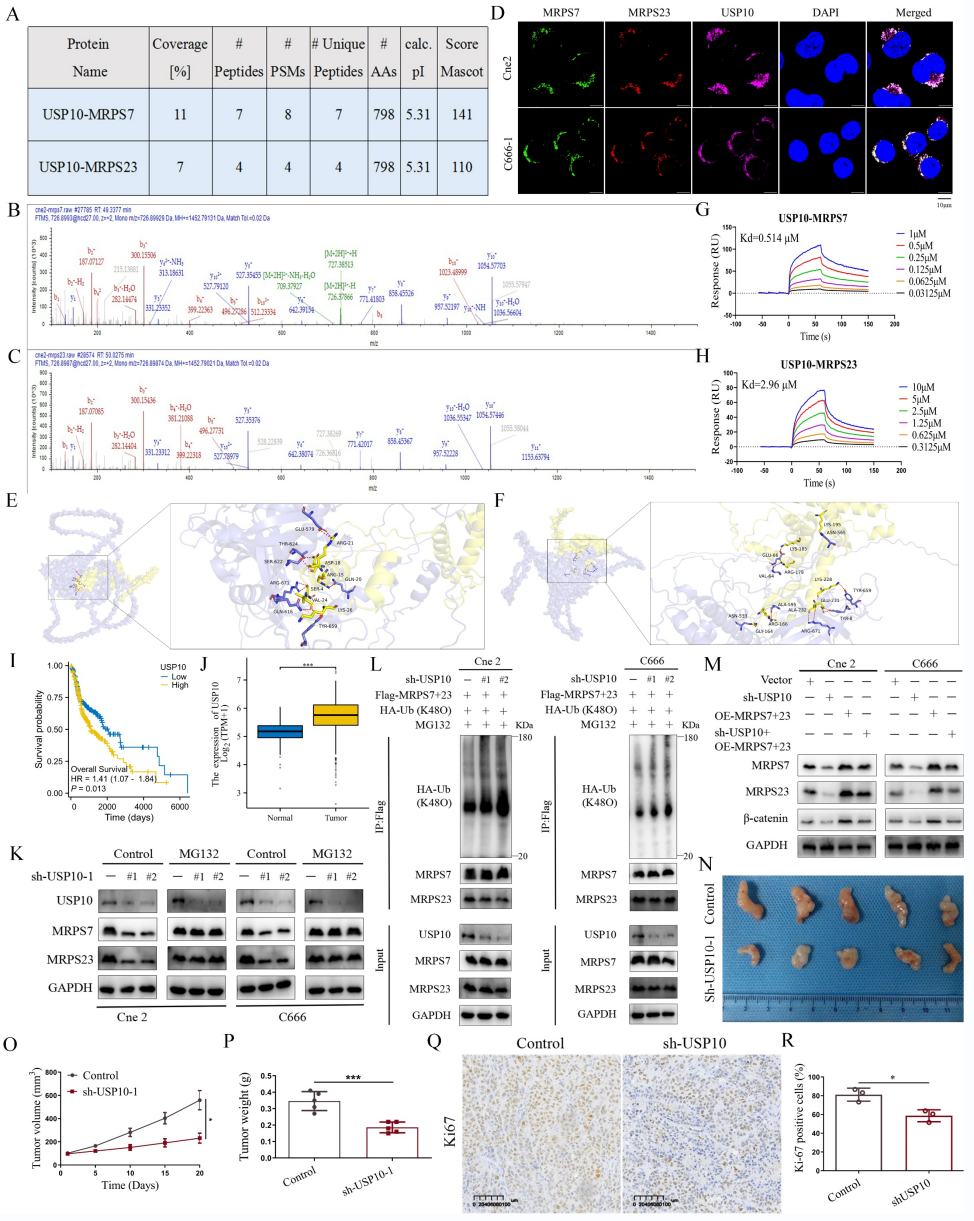
USP10 depletion reduces expression of MRPS7 and MRPS23 and suppresses xenograft tumor growth. (A-C) The protein of USP10 co-immunoprecipitated with MRPS7 and MRPS23 was verified by mass spectrometry. (D) The co-localization of MRPS7 (green), MRPS23 (red) and USP10 (purple) detected by immunofluorescence assay in NPC cells. The scale bar is 10 μm. (E) The binding interface between MRPS7 (yellow) and USP10 (purple) was predicted using computational molecular docking. (F) The binding interface between MRPS23 (yellow) and USP10 (purple) was predicted using computational molecular docking. (G) Direct interaction between USP10 and MRPS7 measured by surface plasmon resonance. (H) Direct interaction between USP10 and MRPS23 measured by surface plasmon resonance. (I) OS curves of low and high expressed of USP10. (J) Bioinformatic analysis of TCGA data revealed differential expression of USP10 in HNSC tumors compared to normal tissues. (K) Western blot analysis of MRPS7, MRPS23 and USP10 protein levels in NPC cells with or without knockdown of USP10 after the treatment with MG132. (L) Following co-transfection with HA-Ub-K48O and either an empty vector or Flag-MRPS7/23 plasmids, NPC cells were treated with MG132 and lysates were immunoprecipitated under denaturing conditions with the specified antibodies. (M) Western blot analysis was conducted to assess MRPS7, MRPS23, and β-catenin expression in USP10-knockdown NPC cells, with or without concomitant overexpression of MRPS7 and MRPS23. (N) The photo of excised Cne2 tumors. (O) Tumor volumetric measurements were evaluated at predetermined intervals. (P) The measurement of tumor weight in excised Cne2 tumors. (Q-R) Ki67 staining and expression of tumor tissue. *p< 0.05 and ***p < 0.001.

The above findings suggest that MRPS7 and MRPS23 play critical roles in tumor growth and cisplatin chemosensitivity in NPC. To further elucidate the regulatory mechanisms governing MRPS7 and MRPS23 expression, we investigated potential upstream binding partners of MRPS7 and MRPS23. Strikingly, IP-MS analysis identified USP10 as a high-confidence interactor with both MRPS7 and MRPS23, supported by its robust Mascot score, extensive peptide coverage, and a high number of unique peptides (Fig. 7A-C). Furthermore, this interaction was supported by the observed colocalization of MRPS7, MRPS23, and USP10 in NPC cells using immunofluorescence staining (Fig. 7D). To assess the possibility of a direct interaction, we performed molecular docking simulations, which revealed structurally complementary interfaces between MRPS7/MRPS23 and USP10, implying a stable binding mode (Fig. 7E-F). More importantly, SPR assays quantified high-affinity binding between USP10 and MRPS7 or MRPS23, with dissociation constants (KD value) of 0.514 μΜ and 2.96 μΜ, respectively (Fig. 7G-H). Functionally, knockdown of USP10 notably decreased MRPS7 and MRPS23 protein levels, whereas treatment with the proteasome inhibitor MG132 reversed this effect, suggesting that USP10 positively regulates the expression of MRPS7 and MRPS23 via the ubiquitin-proteasome pathway (Fig. 7K). Additionally, knockdown of USP10 specifically promoted K48-linked ubiquitination of MRPS7 and MRPS23, without altering K63-linked ubiquitination (Fig. 7L, Fig. S5A-B). To further validate the USP10-MRPS7/MRPS23-β-catenin signaling axis, we overexpressed MRPS7 and MRPS23 in USP10-knockdown cells. Notably, reconstitution of MRPS7 and MRPS23 largely restored β-catenin expression in the absence of USP10 (Fig. 7M). These results establish that USP10 stabilizes β-catenin through the inhibition of ubiquitin-mediated degradation of MRPS7 and MRPS23, defining a functional USP10-MRPS7/MRPS23-β-catenin regulatory pathway. Analysis of TCGA data indicated that USP10 is highly expressed in tumor tissues (Fig. 7J) and is associated with poor prognosis (Fig. 7I). To evaluate the role of USP10 in tumor growth *in vivo*, we utilized a subcutaneous xenograft mouse model. Knockdown of USP10 significantly suppressed tumor growth, as evidenced by reduced tumor volume and mass (Fig. 7N-P), and decreased Ki-67 staining, indicating lower proliferative activity (Fig. 7Q-R). These results demonstrate that USP10 depletion inhibits both MRPS7/MRPS23 expression and tumor growth in NPC.

### Spautin-1, a potent USP10 inhibitor, synergizes with cisplatin to suppress tumor growth and metastasis in NPC

**Figure 8:**
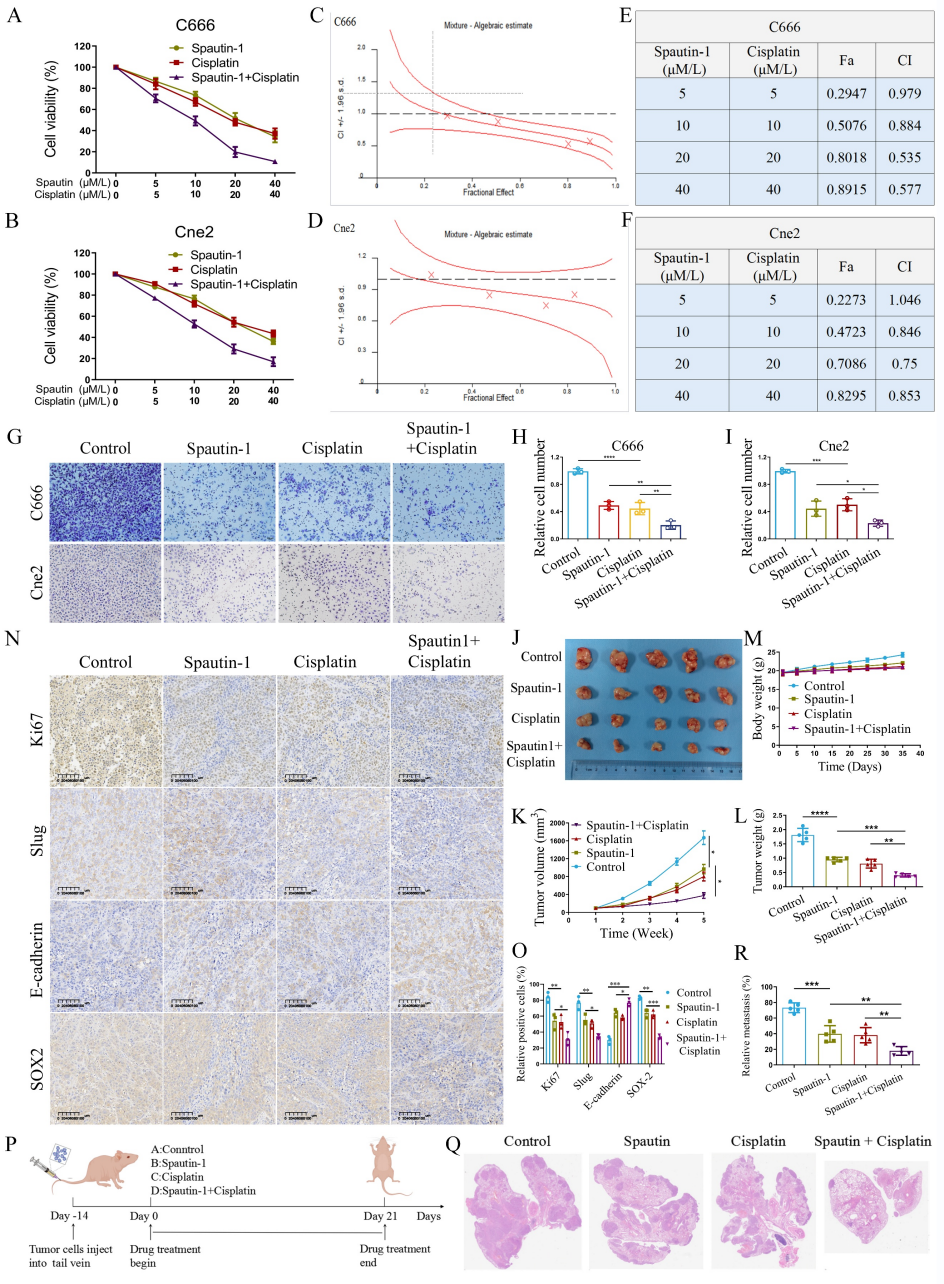
Therapeutic targeting of USP10 by spautin-1 potentiates cisplatin efficacy to suppress tumor growth and metastasis in NPC. (A-B) Cne2 and C666 cells were incubated with Spautin-1, cisplatin, or combination therapy at graded concentrations for 48 h, with viability determined by CCK-8 assay. (C-D) Synergistic effects of Spautin-1/Cisplatin combination in Cne2 and C666 cells were quantitatively analyzed using CalcuSyn software. (E-F) Dose-dependent combination indexes (CIs) and fraction affected (Fa) values for Spautin-1/Cisplatin treatment in both cell lines are presented. (G-I) The Spautin-1/Cisplatin combination significantly suppressed the migratory capacity of NPC cells. (J) Images of resected Cne2 xenograft tumors. (K) Tumor growth kinetics were monitored at specified time points. (L) Tumor weight of excised Cne2 tumors. (M) The mice were weighted on the indicated days. (N) Ki67, slug, E-cadherin and SOX2 staining and expression of tumor tissue. (O) The relative expression of Ki67, slug, E-cadherin and SOX2 in tumor tissue. (P) Schematic diagram of spautin-1 and cisplatin treatment on NPC lung metastasis. (Q-R) H&E-stained lung tissue of lung metastasis model, with subsequent quantification of microscopically detectable metastatic foci. *p< 0.05, **p < 0.01, ***p < 0.001, and ****p < 0.0001.

Given that knockdown of USP10 reduced the expression of MRPS7 and MRPS23, and considering the lack of potent antagonists targeting MRPS7 and MRPS23 directly, we investigated spautin-1, a well-characterized USP10 inhibitor with demonstrated anti-tumor effects, as a potential therapeutic agent. Therefore, we further explored whether Spautin-1 had a synergistic effect with cisplatin in anti-tumor growth. To evaluate whether Spautin-1 synergizes with cisplatin to inhibit tumor growth, we performed CCK-8 assays. The results revealed that the combination of Spautin-1 and cisplatin significantly enhanced cell inhibition compared to either treatment alone (Fig. 8A-B). To quantify the synergistic effects, we calculated combination index (CI) values using CalcuSyn software for various dose combinations (Fig. 8C-D). Our data showed that the CI values of each dose combination were both lower than 1 in C666 cell line (Fig. 8E). Moreover, these dose combination were all both lower than 1 in Cne2 cell lines: 10 μM/L spautin-1 and 10 μM/L cisplatin, 20 μM/L spautin-1 and 20 μM/L cisplatin, 40μM/L spautin-1 and 40 μM/L cisplatin (Fig. 8F). The CI values lower than 1 indicated strong synergistic effects in C666 and Cne2 cell lines (Fig. 8E-F). Notably, the combination of 20 μM/L Spautin-1 and 20 μM/L cisplatin exhibited particularly pronounced synergy, with CI values of 0.535 in C666 cells and 0.75 in Cne2 cells (Fig. 8E-F). These findings demonstrate that 20 μM/L Spautin-1 synergizes with 20 μM/L cisplatin to enhance anti-tumor efficacy. Based on this synergistic effect, this combination was selected for further investigation. Furthermore, western blot analysis further revealed that spautin-1 downregulated the protein expression of USP10, MRPS7, and MRPS23, whereas MG132 treatment restored MRPS7 and MRPS23 expression in NPC cells (Fig. S6A-B). Furthermore, overexpression of MRPS7 and MRPS23 partially rescued the downregulation of β-catenin induced by Spautin-1, indicating that USP10 regulates β-catenin expression through MRPS7 and MRPS23 (Fig. S6C). To investigate whether Spautin-1 and cisplatin synergistically inhibit cell migration, we performed transwell assays. The results revealed that the combination of 20 μM/L Spautin-1 and 20 μM/L cisplatin significantly reduced cell migration compared to either treatment alone (Fig. 8G-I). Building on these molecular insights, we evaluated the therapeutic potential of Spautin-1 and cisplatin *in vivo*. Spautin-1 treatment potentiated cisplatin efficacy in subcutaneous xenografts, as evidenced by significantly diminished tumor volume and weight relative to single-agent cisplatin treatment (Fig. 8J-L), while with no significant alterations in mice body weight (Fig. 8M). Relative to control animals, the tumor weight was reduced by 40.01% with the treatment of spautin-1. Notably, the Spautin-1/cisplatin combination therapy demonstrated significantly enhanced tumor growth suppression, the tumor weight was reduced by 74.75% compared with control group. Immunohistochemical analysis further revealed a significant decrease in Ki-67, Slug and SOX2 expression, accompanied by an increase in E-cadherin levels (Fig. 8N-O). These findings suggest that the combination treatment effectively reverses EMT and cancer cell stemness, a key driver of tumor progression and metastasis. Furthermore, in the lung metastasis model, both Spautin-1 and cisplatin alone significantly reduced the number of metastatic lung nodules. Strikingly, the combination treatment resulted in an even more pronounced decrease in metastatic burden (Fig. 8P-R). Collectively, these results demonstrate the synergistic anti-tumor effects of Spautin-1 and cisplatin to suppress tumor growth and metastasis *in vitro* and *in vivo*.

### MRPS7 and MRPS23 predict poor prognosis and chemoresistance in nasopharyngeal carcinoma

**Figure 9:**
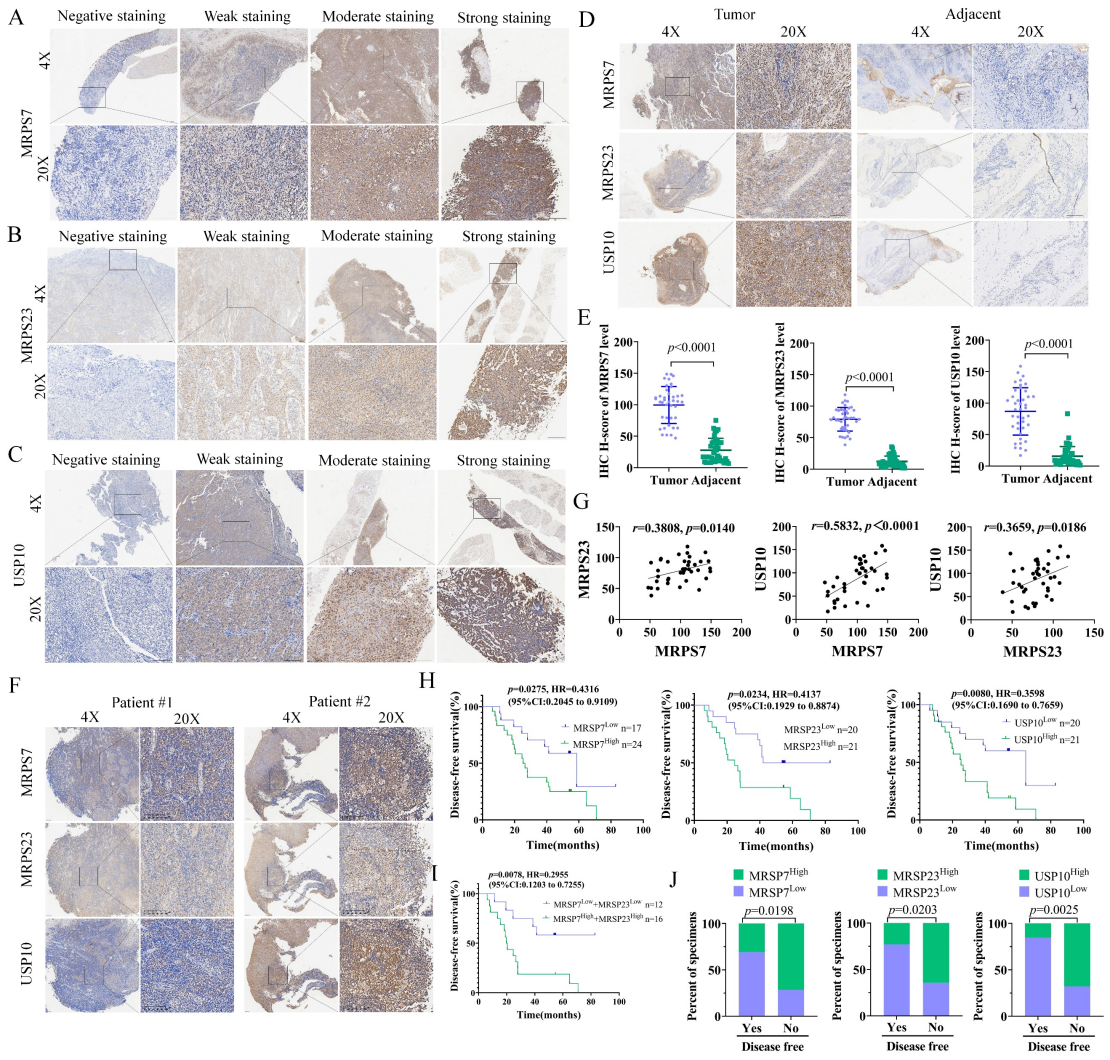
MRPS7, MRPS23, and USP10 expression predicts poor outcomes and chemoresistance in nasopharyngeal carcinoma. (A-C) Immunohistochemical (IHC) staining showing different intensities of MRPS7, MRPS23, and USP10 expression in NPC tissues. Scale bar, 100 μm. (D-E) Comparative IHC analysis of MRPS7, MRPS23, and USP10 expression in tumor versus adjacent non-tumor tissues (n = 41). Scale bar, 100 μm. (F) IHC images of MRPS7, MRPS23, and USP10 in NPC patient samples. Scale bar, 100 μm. (G) Correlation analysis of MRPS7 with MRPS23, MRPS7 with USP10, and MRPS23 with USP10 in NPC tissues (n = 41). (H-I) Kaplan-Meier survival curves demonstrating that high expression of MRPS7, MRPS23, and USP10 is associated with shorter disease-free survival. (J) MRPS7, MRPS23 and USP10 expression, as evaluated by IHC staining, was associated with the response to cisplatin treatment.

To further evaluate the clinical relevance of MRPS7 and MRPS23 in NPC, we performed IHC staining on tumor specimens from 41 patients who had received cisplatin (DDP)-based therapy (Table S1-3). In NPC tissues, MRPS7 and MRPS23 were predominantly expressed in the cytoplasm. Besides, USP10 was detected in both the cytoplasm and nucleoli. Samples were categorized into four groups based on staining intensity: negative, weak, moderate, and strong expression (Fig. 9A-C).

A significant upregulation of MRPS7, MRPS23, and USP10 was observed in tumor tissues relative to matched adjacent non-tumor tissues (Fig. 9D-E). Moreover, MRPS7 and MRPS23 expression levels were positively correlated with USP10 expression (Fig. 9F-G). Besides, Kaplan-Meier survival analysis demonstrated that high expression of these proteins correlated with shorter disease-free survival (DFS) (Fig. 9H). Notably, the concurrent high expression of MRPS7 and MRPS23 exhibited an even stronger correlation with shorter disease-free survival (DFS) (Fig. 9I). Analysis of clinical data revealed that elevated levels of MRPS7, MRPS23, and USP10 were significantly linked to reduced DFS (Fig. 9J). Collectively, these findings indicate that MRPS7 and MRPS23 expression in NPC is closely associated with poor prognosis, cisplatin chemoresistance, and coordinated expression with USP10, highlighting their potential clinical significance and mechanistic interplay in NPC progression.

## Discussion

NPC, an Epstein-Barr virus-associated malignancy with a distinct geographical prevalence in Southern China, represents a formidable clinical challenge due to its characteristic metastatic propensity and intrinsic chemoresistance^45^-^49^. To uncover the molecular mechanisms driving NPC progression, we conducted a comprehensive analysis of transcriptomic datasets and clinical specimens, revealing that mitochondrial ribosomal proteins MRPS7 and MRPS23 are significantly overexpressed in NPC patients. Importantly, a strong positive correlation (high R-value) was observed between MRPS7 and MRPS23 expression, suggesting a potential functional interplay between the two proteins. Fortunately, the functional validation experiments demonstrated that individual knockdown of either MRPS7 or MRPS23 markedly inhibited cell proliferation in both *in vitro* and *in vivo* experimental models. Strikingly, dual knockdown of MRPS7 and MRPS23 resulted in synergistic anti-tumor effects, mediated through the suppression of β-catenin signaling. This suppression led to the inhibition of EMT and cancer stem cell properties, highlighting the cooperative role of MRPS7 and MRPS23 in NPC pathogenesis. These findings establish MRPS7 and MRPS23 as novel co-expressed genes that critically contribute to the malignant progression of NPC. Accumulating preclinical and clinical evidence has demonstrated that the co-activation of EMT programs and cancer stemness properties represents a pivotal mechanism underlying concurrent chemoresistance and metastatic potential in cancer^50^-^53^. Our findings revealed that the knockdown of MRPS7 and MRPS23 synergistically enhanced the anti-tumor efficacy of cisplatin in NPC. Further investigation into the regulatory mechanisms revealed that USP10 acts as a key regulator of MRPS7 and MRPS23 expression. Notably, pharmacological inhibition of USP10 using Spautin-1 synergistically enhanced the anti-tumor efficacy of cisplatin, significantly suppressing tumor growth in preclinical models. This discovery highlights the therapeutic potential of targeting the USP10-MRPS7/MRPS23-β-catenin axis to overcome chemoresistance in NPC. In summary, our study identifies MRPS7 and MRPS23 as pivotal oncogenic drivers that promote tumor growth, metastasis, and chemoresistance in NPC. Furthermore, we propose a novel therapeutic strategy combining USP10 inhibition with cisplatin to enhance treatment efficacy, offering a promising approach for improving outcomes in NPC patients.

MRPSs, encoded by nuclear DNA, synthesized in the cytoplasmic compartment, yet primarily exert their functional roles within the mitochondria, encompassing apoptosis, cell cycle, stress response, mitochondrial activity, transcription and maintenance^54^-^56^. Besides, MRPSs have been increasingly associated with oncogenesis, particularly in advanced malignancies and metastatic processes spanning diverse cancer subtypes, where they critically regulate cellular survival and disease progression^57^-^59^. The oncogenic role of MRPS23 was first found in metastatic progression, with its upregulation identified in recurrent cervical carcinomas exhibiting lymph node metastasis and enhanced proliferative and invasive capacities^24^. Elevated expression of MRPS23 demonstrates a robust correlation with poorly differentiated, clinically aggressive breast carcinomas^60^. Post-translational modifications of MRPS23, particularly arginine and lysine methylation, facilitate metastatic progression in breast cancer by modulating oxidative phosphorylation pathways^61^. Besides, the long non-coding RNA HIF1A-AS2 facilitates tumor progression and confers paclitaxel resistance in triple-negative breast cancer through modulation of MRPS23 protein expression^24^. Both MRPS6 and MRPS23 exhibit significant dysregulation in breast malignancies, where they critically influence oncogenic pathways and cellular transformation processes^16^. In glioma, MRPS23 has emerged as a novel prognostic biomarker and functional driver of tumor progression, underscoring its potential as a therapeutic target^21^. MRPS23 overexpression correlates with advanced tumor stage and reduced survival, although its direct role in metastatic dissemination remains unclear. MRPS23 has been identified as one of twelve functionally significant RNA-binding proteins demonstrating prognostic relevance in colorectal carcinoma, though intriguingly, its elevated expression correlates with improved clinical outcomes^23^. However, the mechanistic basis for this apparent low-risk profile remains poorly understood. These divergent findings highlight the conserved oncogenic contributions of MRPS23 across various cancer types, while emphasizing its tissue-specific and mechanism-specific roles in tumor progression. Despite these advances, the role of MRPS23 in NPC remains unexplored. MRPS7 was identified as a potential crucial gene in the Malignant progression of osteosarcoma^62^. Besides, knockdown of MRPS7 proved selectively toxic to diffuse large B-cell lymphoma cell lines, which suggested MRPS7 emerges as a promising molecular target for therapeutic intervention in diffuse large B-cell lymphoma^63^. Moreover, MRPS7 was predominantly detected in epithelial breast cancer cells but was notably absent or minimally expressed in neighboring tumor stromal cells, which may participate the malignant progression of breast cancer^20^. Compared with MRPS23, the functional role of MRPS7 in cancer remains poorly characterized. Novel therapeutic strategies targeting mitochondrial biogenesis and translation machinery in cancer warrant further development. Our study found that MRPS7 and MRPS23, hub genes of MRPSs, elevated expression levels of both MRPS7 and MRPS23 were observed in the HNSC TCGA dataset and correlated with significantly worse OS. Importantly, the high expression of MRPS7 and MRPS23 were also validated in GSE61218 database of NPC. Here, our study revealed that knockdown expression of MRPS7 and MRPS23 exhibited an anti-tumor effect and have a synergistic effect with cisplatin, further investigation into the regulatory mechanisms revealed that USP10 acts as a key regulator of MRPS7 and MRPS23 expression. This discovery highlights the therapeutic potential of targeting the USP10-MRPS7/MRPS23-β-catenin axis to overcome chemoresistance in NPC. These findings suggest MRPs as promising precision therapeutic targets for advanced-stage malignancies that currently lack effective treatment options.

USP10, as a ubiquitin-specific protease, serves as a critical regulator in deubiquitinating substrates and stabilizing intracellular proteins, thereby regulating diverse cellular processes^64^. USP10 has emerged as a central driver of oncogenesis across diverse malignancies, where it coordinates critical signaling cascades that regulate hallmark cancer processes, including uncontrolled proliferation, stemness maintenance, metastatic dissemination, and therapeutic resistance^65^-^68^. Mechanistically, USP10 drives tumor progression through multiple molecular axes, each contributing to distinct oncogenic processes. As for metabolic reprogramming, SPICE1 facilitates osteosarcoma progression by enhancing USP10-mediated deubiquitination and stabilization of FASN, thereby fueling lipid biosynthesis and tumor growth^69^. As for DNA damage repair and chemoresistance: The USP10/XAB2/ANXA2 axis promotes DNA damage repair, enhancing chemoresistance to oxaliplatin in colorectal cancer^37^. Moreover, Pseudokinase TRIB3 promotes multiple myeloma progression by stabilizing SSRP1 through USP10-mediated deubiquitination, thereby enhancing oncogenic signaling and tumor growth^70^. As for Stemness and Metastasis: USP10 deubiquitinates and stabilizes CD44, driving breast cancer cell proliferation, stemness, and metastatic dissemination^34^, ^35^. Similarly, in colorectal cancer, USP10 sustains cancer stemness by enabling super-competitor signaling, a mechanism critical for tumor niche dominance. In NPC, USP10 has been shown to interact with DCAF7, which recruits USP10 for G3BP1 deubiquitylation, facilitating chemoresistance and metastasis^9^. However, the regulatory role of USP10 in modulating MRPS7 and MRPS23 expression in NPC has not been systematically investigated. In this study, we uncovered that USP10 knockdown significantly inhibits tumor growth in NPC. Furthermore, pharmacological inhibition of USP10 using Spautin-1 synergizes with cisplatin to enhance anti-tumor efficacy, suggesting a potential therapeutic strategy for overcoming chemoresistance. These findings highlight the critical role of the USP10-MRPS7/MRPS23 molecular axis as both a key driver of nasopharyngeal carcinoma progression and a novel actionable target for precision oncology approaches.

Cisplatin-based chemotherapies, including induction and concurrent regimens, have significantly improved distant metastasis control and overall survival in NPC patients, establishing cisplatin as the cornerstone of NPC treatment^71^, ^72^. However, despite remarkable reductions in locoregional recurrence, approximately 10% of patients develop chemotherapy resistance and distant metastasis, which remain the primary causes of treatment failure^8^, ^73^. Tackling these challenges mandates systematic dissection of the core molecular circuitry driving metastasis and cisplatin resistance. Recent studies have elucidated some mechanisms underlying NPC metastasis and cisplatin chemoresistance, including USP7-mediated stabilization of KDM5B via the ZBTB16/TOP2A axis^74^, circIPO7-facilitated YBX1 nuclear localization^75^, and SOX1 drives chemoresistance in nasopharyngeal carcinoma by inducing a therapy-refractory cellular state^76^. Despite these advances, the identification of actionable targets to overcome cisplatin resistance remains a critical unmet need.

Emerging evidence indicates that EMT enhances tumor cell migration and invasion while concurrently reducing sensitivity to chemotherapeutic agents, thereby accelerating disease progression^77^-^79^. Concurrently, the acquisition and maintenance of cancer stem cell traits empower a subpopulation of tumor cells to evade cytotoxic stress, regenerate tumor heterogeneity, and drive relapse^80^-^82^. Multiple molecular mechanisms contribute to these processes: for instance, IGF2BP3 stabilizes NOTCH3 mRNA through m6A-dependent suppression of CCR4-NOT-mediated deadenylation, promoting stemness-associated transcriptional programs and metastasis in NPC^83^. Similarly, elevated APSN expression activates Wnt/β-catenin signaling to enhance stemness and EMT, underpinning docetaxel resistance and metastatic progression in prostate cancer^84^. In gastric cancer, hnRNPA2B1 stabilizes lncRNA NEAT1, facilitating CSC properties and chemoresistance via Wnt/β-catenin activation^85^. Furthermore, ThermomiR-377-3p-mediated downregulation of Cirbp is essential for hyperthermia-induced cytotoxicity in both cancer and stem-like cells^86^. While in NPC, the protein C receptor sustains cancer stemness through lipid synthesis activation^87^. Collectively, these mechanisms underscore critical pathways driving chemoresistance and recurrence across malignancies. Our study extends this paradigm by identifying mitochondrial ribosomal proteins MRPS7 and MRPS23 as novel regulators of chemoresistance in NPC. Knockdown of MRPS7 and MRPS23 not only suppresses tumor progression but also synergizes with cisplatin to enhance therapeutic efficacy. Mechanistically, MRPS7/23 depletion destabilizes mitochondrial translation machinery, impairing DNA replication and inhibiting cancer stemness and EMT through suppression of β-catenin signaling. These findings establish MRPS7 and MRPS23 as dual therapeutic targets capable of disrupting both tumorigenic metabolism and chemoresistance networks, offering a promising avenue for precision oncology in NPC.

Rational drug combinations represent a cornerstone strategy in oncology, particularly for aggressive malignancies, with the primary objectives of achieving synergistic efficacy, delaying drug resistance, and minimizing systemic toxicity^88^-^90^. In this study, we demonstrated that dual knockdown of mitochondrial ribosomal proteins MRPS7 and MRPS23 not only exerts intrinsic anti-tumor effects but also synergizes with cisplatin to enhance therapeutic outcomes. Mechanistic interrogation revealed USP10 as an important regulator of MRPS7 and MRPS23 expression, which strongly supports pursuing USP10-targeted interventions as a viable treatment approach. The USP10/USP13 inhibitor spautin-1 has demonstrated broad anti-tumor activity across malignancies, including: Suppression of CD44 stabilization via USP10 inhibition, leading to impaired proliferation, stemness, and metastasis in breast Cancer, disruption of the GSK3β-ULK1 axis to inhibit autophagy and tumor progression in osteosarcoma^91^. Dual modulation of RAF-ERK-mediated glycolysis and SKP2-dependent proteostasis to attenuate tumor growth in glioblastoma^92^. Despite these advances, the therapeutic potential of spautin-1 in NPC remained unexplored. In our study, we demonstrated that pharmacological inhibition of USP10 using spautin-1 synergistically enhances the anti-tumor efficacy of cisplatin, significantly suppressing tumor growth and lung metastasis in preclinical mouse models. Collectively, these findings position spautin-1 as both a standalone therapeutic agent and a chemosensitizer capable of reversing cisplatin resistance in NPC. This dual functionality underscores its potential for clinical translation, offering a novel strategy to improve outcomes in treatment-refractory NPC patients.

Our study identifies MRPS7 and MRPS23 as co-expressed oncogenes that critically contribute to NPC progression. A strong positive correlation between their expression suggests functional interplay, validated by the marked inhibition of cell proliferation upon individual knockdown *in vitro* and *in vivo*. Strikingly, dual knockdown of MRPS7 and MRPS23 synergistically suppressed tumor growth by inhibiting β-catenin signaling, thereby attenuating EMT and cancer stemness. Further mechanistic investigation revealed USP10 as a negative regulator of MRPS7 and MRPS23. Pharmacological inhibition of USP10 using spautin-1 synergized with cisplatin, significantly suppressing tumor growth in preclinical models. This highlights the therapeutic potential of targeting the USP10-MRPS7/MRPS23-β-catenin axis to overcome chemoresistance. In summary, MRPS7 and MRPS23 are pivotal oncogenic drivers in NPC, and their inhibition, particularly in combination with USP10 targeting, offers a promising strategy to enhance cisplatin efficacy and improve patient outcomes.

## Supplementary Material

Supplementary figures and tables.

## Figures and Tables

**Figure 10:**
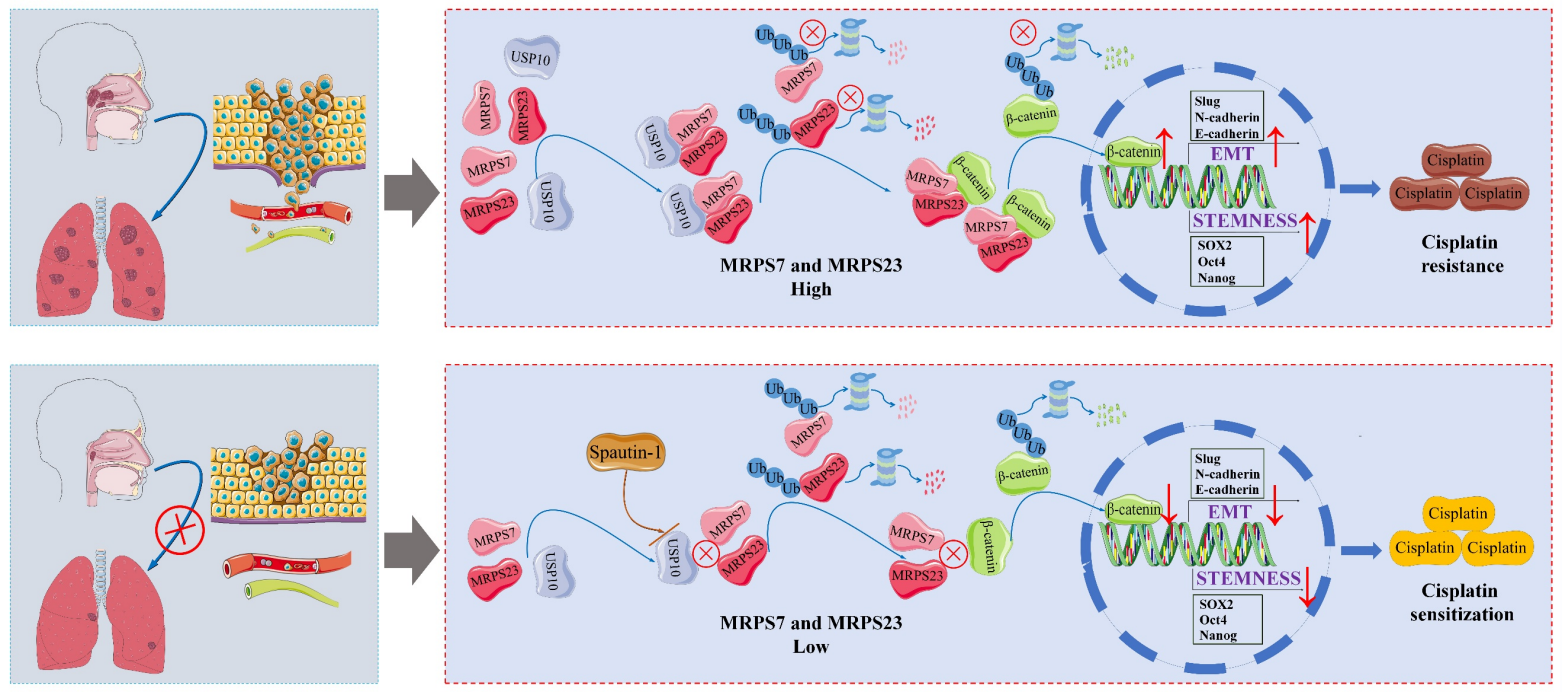
Targeting MRPS7 and MRPS23 potentiates cisplatin efficacy in nasopharyngeal carcinoma by regulating EMT and stemness.

## Abbreviations

NPC: Nasopharyngeal carcinoma
MRPSs: Mitochondrial ribosomal small subunit proteins
HNSC: head and neck squamous cell carcinoma
MRPS7: Mitochondrial Ribosomal Protein S7
shRNA: Short Hairpin RNA
TPF: docetaxel-cisplatin-5-fluorouracil
t-SNE: t-Distributed stochastic neighbor embedding
USP10: Ubiquitin-specific peptidase 10
MRPS23: Mitochondrial Ribosomal Protein S23
GP: gemcitabine-cisplatin
USP: Ubiquitin-specific protease
OS: Overall Survival
CI: combination index
CSC: cancer stem cell
TISCH: Tumor Immune Single-Cell Hub
Fa: fraction affected
LASSO: least absolute shrinkage and selection operators
ScRNA-seq: single-cell RNA-sequence
FBS: Fetal Bovine Serum
ADCs: antibody-drug conjugates
PBS: Phosphate-Buffered Saline
TCGA: The Cancer Genomic Atlas
IP: immunoprecipitated
Co-IP: co-immunoprecipitation
EDC: 1-ethyl-3-(3-dimethylaminopropyl)carbodiimide
RT-qPCR: Reverse Transcription-Quantitative Polymerase Chain Reaction
GEO: Gene Expression Omnibus
NHS: N-hydroxysuccinimide
H&E: hematoxylin & eosin
IHC: Immunohistochemical
PFA: paraformaldehyde
DTT: dithiothreitol
EMT: Epithelial-Mesenchymal Transition
CCK-8: Cell Counting Kit 8
SPR: Surface plasmon resonance
