# Supplementary material for: Co-targeting MRPS7-23 synergistically enhances cisplatin efficacy to suppress nasopharyngeal carcinoma growth and metastasis
Source: Int J Biol Sci. 2026 Jan 1;22(2):970–94. doi: 10.7150/ijbs.115523 (PMC12781175; doi:10.7150/ijbs.115523)
Supplement: Supplementary file 1 — Supplementary figures and tables. [file ijbsv22p0970s1.pdf]

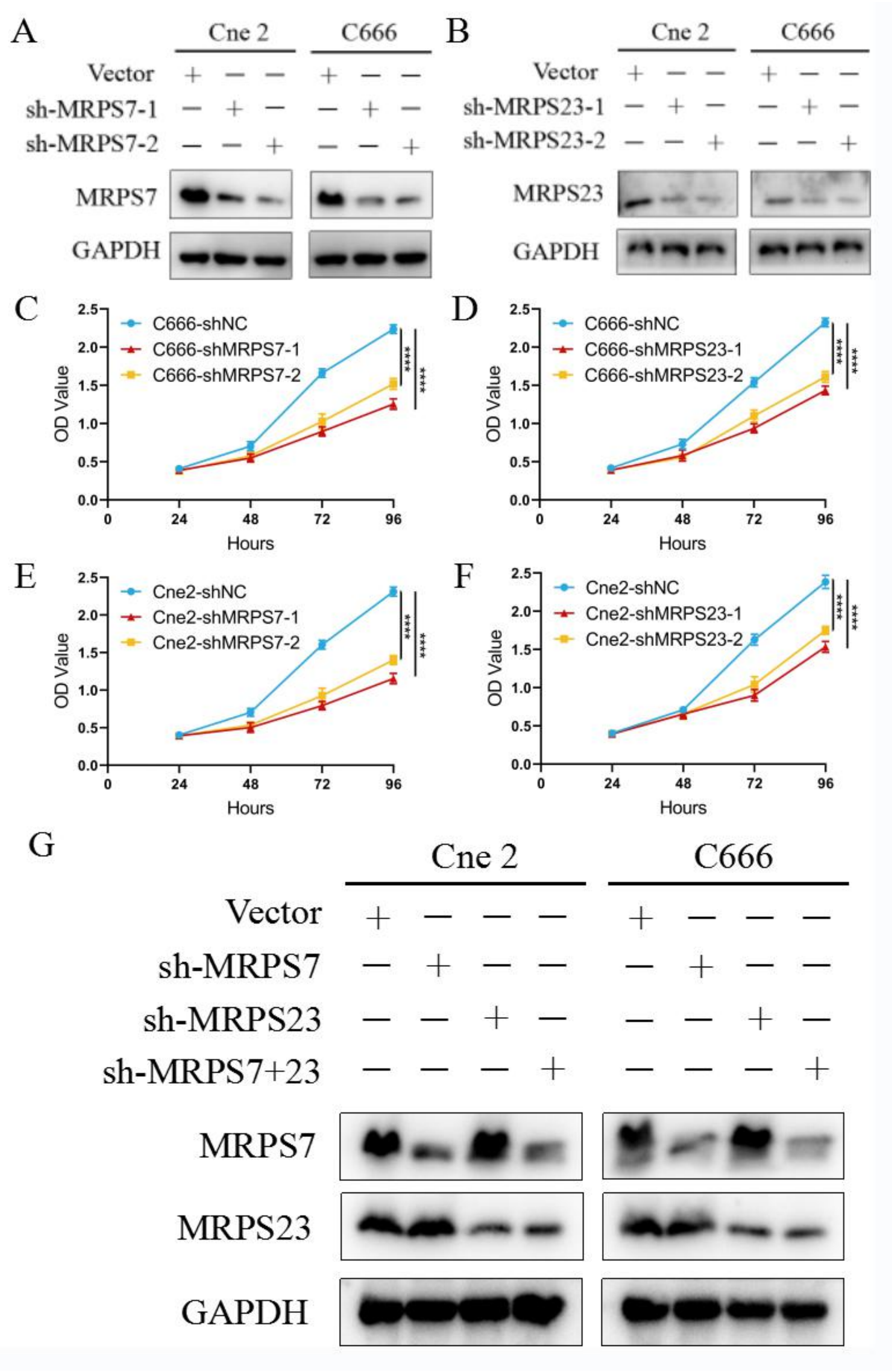

Figure S1. MRPS7 and MRPS23 knockdown suppresses NPC cell growth. (A) Western blot analysis confirming successful knockdown of MRPS7 in Cne2 and C666 cells. (B) Western blot analysis confirming successful knockdown of MRPS23. (C–F) Growth curve analysis showing that knockdown of MRPS7 or MRPS23 inhibited NPC cell proliferation. (G) Western blot analysis confirming successful double knockdown of MRPS7 and MRPS23. **** $p < 0.0001$.

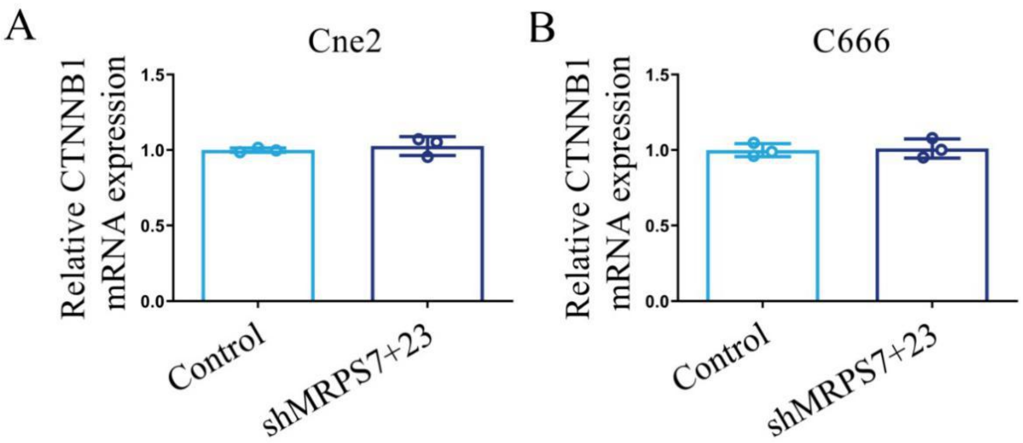

Figure S2. MRPS7 and MRPS23 knockdown does not affect CTNNB1 mRNA expression. (A-B) qRT-PCR analysis showing β-catenin mRNA levels in NPC cells with or without MRPS7/MRPS23 knockdown.

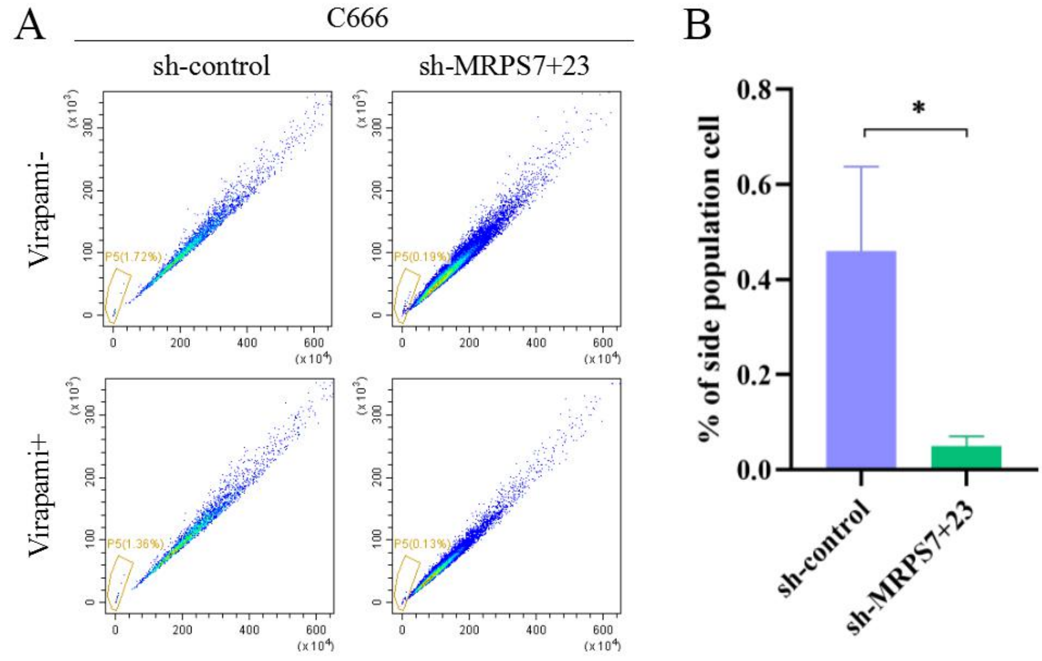

Figure S3. MRPS7 and MRPS23 knockdown reduces stem-like properties of NPC cells. (A) Flow cytometry analysis of side population (SP) cells in NPC cells with MRPS7 or MRPS23 knockdown. (B) Quantification of SP cell proportions. * $p < 0.05$.

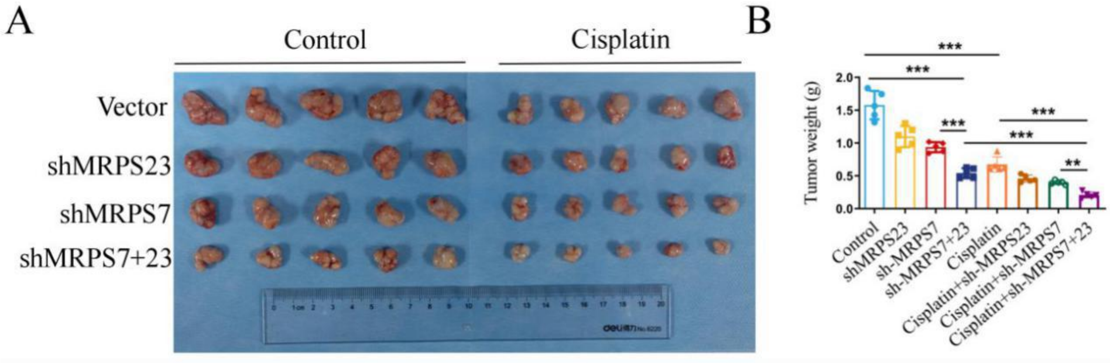

Figure S4. MRPS7 and MRPS23 knockdown enhances cisplatin chemosensitivity and suppresses tumor growth in vivo. (A) Representative images of excised tumors from nude mice xenografted with Cne2 cells. (B) Tumor weight of excised Cne2 tumors. ** $p < 0.01$, *** $p < 0.001$.

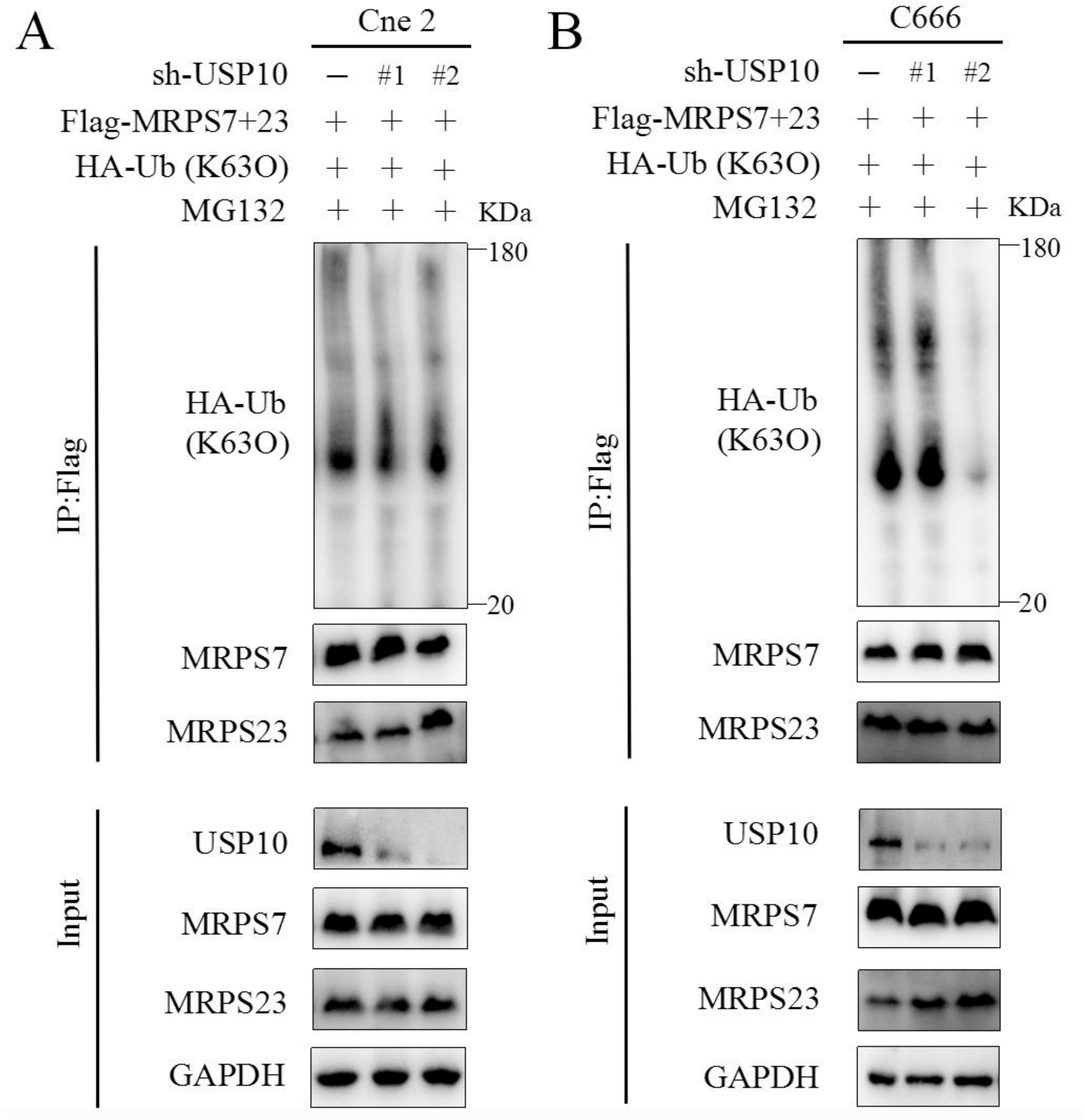

Figure S5. Knockout of USP10 did not alter the K63-linked ubiquitination of MRPS7 and MRPS23. (A-B) Following co-transfection with HA-Ub-K63O and either an empty vector or Flag-MRPS7/23 plasmids, NPC cells were treated with MG132 and lysates were immunoprecipitated under denaturing conditions with the specified antibodies.

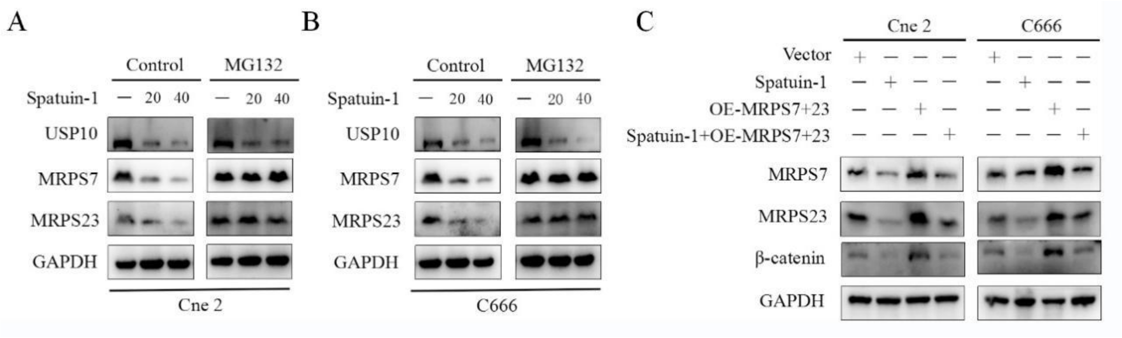

Figure S6. Spautin-1 destabilizes β-catenin by downregulating USP10 and subsequently reducing MRPS7/MRPS23 expression. (A-B) Western blot analysis of MRPS7, MRPS23, and USP10 protein levels in NPC cells treated with Spautin-1 at different doses, with or without MG132. (C) Western blot analysis of MRPS7, MRPS23, and β-catenin protein levels in NPC cells overexpressing MRPS7 and MRPS23, following treatment with spautin-1.

Table S1. Correlations between MRPS7 expression levels and clinical features in nasopharyngeal carcinoma patients.

Characteristic	Number of patients (%)		
	Low expression group (n = 17)	High expression group (n = 24)	*P*-value*
Gender			
Male	13	16	0.7289
Female	4	8	
Age (years old)			
< 60	15	23	0.5598
≥ 60	2	1	
TNM stage†			
I/II	0	0	>0.9999
III/IV	17	24	
Metastasis			
Yes	8	20	0.0198
No	9	4	
Death			
Yes	0	3	0.2537
No	17	21	

* Pearson $\chi 2$ test was used to compute the P-value.

† All patients were restaged based on the 7th edition of the AJCC Cancer Staging Manual.

Table S2. Correlations between MRPS23 expression levels and clinical features in nasopharyngeal carcinoma patients.

Characteristic	Number of patients (%)		*P*-value*
	Low expression group (n = 20)	High expression group (n = 21)	
Gender			
Male	16	13	0.3058
Female	4	8	
Age (years old)			
< 60	18	20	0.6060
≥ 60	2	1	
TNM stage†			
I/II	0	0	>0.9999
III/IV	20	21	
Metastasis			
Yes	10	18	0.0203
No	10	3	
Death			
Yes	1	2	>0.9999
No	19	19	

* Pearson $\chi 2$ test was used to compute the P-value.

† All patients were restaged based on the 7th edition of the AJCC Cancer Staging Manual.

Table S3. Correlations between USP10 expression levels and clinical features in nasopharyngeal carcinoma patients.

Characteristic	Number of patients (%)		*P*-value*
	Low expression group (n = 20)	High expression group (n = 21)	
Gender			
Male	12	17	0.1809
Female	8	4	
Age (years old)			
< 60	18	20	0.6060
≥ 60	2	1	
TNM stage†			
I/II	0	0	>0.9999
III/IV	20	21	
Metastasis			
Yes	9	19	0.0025
No	11	2	
Death			
Yes	2	1	0.6060
No	18	20	

* Pearson $\chi 2$ test was used to compute the P-value.

† All patients were restaged based on the 7th edition of the AJCC Cancer Staging Manual.
